# Negative symptoms in children and adolescents with early-onset psychosis and at clinical high-risk for psychosis: systematic review and meta-analysis

**DOI:** 10.1192/bjp.2022.203

**Published:** 2023-07

**Authors:** Gonzalo Salazar de Pablo, Ana Catalan, Julio Vaquerizo Serrano, Borja Pedruzo, Luis Alameda, Veronica Sandroni, Alvaro Armendariz, Victoria Rodriguez, Celso Arango, Carmen Moreno, Johnny Downs, Chris Abbott, Jae Il Shin, Marco Solmi, Paolo Fusar-Poli, Christoph U. Correll

**Affiliations:** Department of Child and Adolescent Psychiatry, Institute of Psychiatry, Psychology & Neuroscience, King's College London, London, UK; Early Psychosis: Interventions and Clinical Detection (EPIC) Lab, Department of Psychosis Studies, Institute of Psychiatry, Psychology & Neuroscience, King's College London, London, UK; Child and Adolescent Mental Health Services, South London and Maudsley NHS Foundation Trust, London, UK; and Department of Child and Adolescent Psychiatry, Institute of Psychiatry and Mental Health, Hospital General Universitario Gregorio Marañón School of Medicine, Universidad Complutense, IiSGM, CIBERSAM, Madrid, Spain; Early Psychosis: Interventions and Clinical Detection (EPIC) Lab, Department of Psychosis Studies, Institute of Psychiatry, Psychology & Neuroscience, King's College London, London, UK; and Mental Health Department, Biocruces Bizkaia Health Research Institute, Basurto University Hospital, Facultad de Medicina y Odontología, Campus de Leioa, University of the Basque Country, UPV/EHU, Vizcaya, Spain; Department of Child and Adolescent Psychiatry, Institute of Psychiatry, Psychology & Neuroscience, King's College London, London, UK; Department of Psychiatry, Basurto University Hospital, Bilbao, Spain; Department of Psychosis Studies, Institute of Psychiatry, Psychology and Neuroscience, King's College London, London, UK; Service of General Psychiatry, Treatment and Early Intervention in Psychosis Program, University Hospital (CHUV), Lausanne, Switzerland; and Centro Investigacion Biomedica en Red de Salud Mental (CIBERSAM), Instituto de Biomedicina de Sevilla (IBIS), Hospital Universitario Virgen del Rocio, Departamento de Psiquiatria, Universidad de Sevilla, Sevilla, Spain; Department of Psychiatry, Faculty of Medicine, Université Paris Cité, Paris, France; and Department of Psychiatry, Groupe Hospitalier Paul Guiraud, Villejuif, France; Department of Psychiatry and Psychology, Hospital Sant Joan de Déu de Barcelona, L'Hospitalet de Llobregat, Barcelona, Spain; Department of Psychosis Studies, Institute of Psychiatry, Psychology and Neuroscience, King's College London, London, UK; Department of Child and Adolescent Psychiatry, Institute of Psychiatry and Mental Health, Hospital General Universitario Gregorio Marañón School of Medicine, Universidad Complutense, IiSGM, CIBERSAM, Madrid, Spain; Child and Adolescent Mental Health Services, South London and Maudsley NHS Foundation Trust, London, UK; Department of Paediatrics, Yonsei University College of Medicine, Seoul, Republic of Korea; Early Psychosis: Interventions and Clinical Detection (EPIC) Lab, Department of Psychosis Studies, Institute of Psychiatry, Psychology & Neuroscience, King's College London, London, UK; Department of Psychiatry, University of Ottawa, Ottawa, Ontario, Canada; Department of Mental Health, The Ottawa Hospital, Ottawa, Ontario, Canada; and Department of Child and Adolescent Psychiatry, Charité Universitätsmedizin, Berlin, Germany; Early Psychosis: Interventions and Clinical Detection (EPIC) Lab, Department of Psychosis Studies, Institute of Psychiatry, Psychology & Neuroscience, King's College London, London, UK; Department of Brain and Behavioral Sciences, University of Pavia, Pavia, Italy; OASIS service, South London and Maudsley NHS Foundation Trust, London, UK; and National Institute for Health Research, Maudsley Biomedical Research Centre, South London and Maudsley NHS Foundation Trust, London, UK; Department of Child and Adolescent Psychiatry, Charité Universitätsmedizin, Berlin, Germany; Department of Psychiatry, Northwell Health, The Zucker Hillside Hospital, Glen Oaks, New York, USA; Department of Psychiatry and Molecular Medicine, Zucker School of Medicine at Hofstra/ Northwell, Hempstead, New York, USA; and Center for Psychiatric Neuroscience, The Feinstein Institutes for Medical Research, Manhasset, New York, USA

**Keywords:** Early-onset psychosis, psychotic disorders, meta-analysis, clinical high risk of psychosis, prevention

## Abstract

**Background:**

Early-onset psychosis (EOP) refers to the development of a first episode of psychosis before 18 years of age. Individuals at clinical high risk for psychosis (CHR-P) include adolescents and young adults, although most evidence has focused on adults. Negative symptoms are important prognostic indicators in psychosis. However, research focusing on children and adolescents is limited.

**Aims:**

To provide meta-analytical evidence and a comprehensive review of the status and advances in the diagnosis, prognosis and treatment of negative symptoms in children and adolescents with EOP and at CHR-P.

**Method:**

PRISMA/MOOSE-compliant systematic review (PROSPERO: CRD42022360925) from inception to 18 August 2022, in any language, to identify individual studies conducted in EOP/CHR-P children and adolescents (mean age <18 years) providing findings on negative symptoms. Findings were systematically appraised. Random-effects meta-analyses were performed on the prevalence of negative symptoms, carrying out sensitivity analyses, heterogeneity analyses, publication bias assessment and quality assessment using the Newcastle–Ottawa Scale.

**Results:**

Of 3289 articles, 133 were included (*n* = 6776 EOP, mean age 15.3 years (s.d. = 1.6), males = 56.1%; *n* = 2138 CHR-P, mean age 16.1 years (s.d. = 1.0), males = 48.6%). There were negative symptoms in 60.8% (95% CI 46.4%–75.2%) of the children and adolescents with EOP and 79.6% (95% CI 66.3–92.9%) of those at CHR-P. Prevalence and severity of negative symptoms were associated with poor clinical, functional and intervention outcomes in both groups. Different interventions were piloted, with variable results requiring further replication.

**Conclusions:**

Negative symptoms are common in children and adolescents at early stages of psychosis, particularly in those at CHR-P, and are associated with poor outcomes. Future intervention research is required so that evidence-based treatments will become available.

Early-onset psychosis (EOP) refers to the development of a first episode of psychosis before the age of 18 years.^1^ EOP is characterised by a high frequency of negative symptoms.^2^ Negative symptoms are defined as a reduction of normal functions related to either motivation and interest (e.g. avolition, anhedonia and asociality) or expressive functions (e.g. blunted affect and alogia)^3^ and can be evaluated categorically based on their presence/absence or continuously based on their severity. Younger age at onset is associated with more negative symptoms at follow-up,^4^ and the severity of negative symptoms is associated with several poor outcomes.^5–7^ Negative symptoms are challenging to identify in young people,^8^ and over 60% of individuals with EOP experience ‘poor’ long-term outcomes and unmet therapeutic needs.^9^ Furthermore, the prevalence of negative symptoms in EOP and their effect on prognosis remains unclear.^6^

Prior research in populations at clinical high risk for psychosis (CHR-P) typically includes adolescents and young adults^10^ from different risk groups, including ultra-high risk criteria (Supplementary eTable 1, available at https://doi.org/10.1192/bjp.2022.203) and basic symptom criteria (Supplementary eTable 2). However, most of the evidence examining negative symptoms focuses on adult or mixed CHR-P samples, with relatively little research focusing on children and adolescents.^11,12^ However, in children and adolescents at CHR-P, negative symptoms have been found to be clinically relevant and sometimes predominant psychopathologically.^13^ Negative symptoms have also been associated with poor outcomes and poor recovery levels in this population.^14^

To our knowledge, no previous meta-analysis has evaluated the prevalence of negative symptoms in children and adolescents with EOP or at CHR-P or investigated the influence of moderating factors, such as gender, age or study design, on the prevalence of negative symptoms. From a diagnostic and prognostic perspective, previous systematic reviews and meta-analyses have variously examined the link between negative symptoms and functioning in individuals at CHR-P,^15^ the association between cannabis and nicotine use and negative symptoms,^16^ the association between the duration of untreated psychosis and negative symptoms^17^ and the relationship between depressive symptoms and negative symptoms.^18^ These studies have been limited in scope: they have just examined correlates of negative symptoms or outcomes associated with negative symptoms;^15–18^ none of these studies focused on children and adolescents; none comprehensively summarised the available evidence or provided methodological and research agenda recommendations to advance the field.

Previous meta-analyses have evaluated the efficacy of antipsychotic medications for the treatment of psychotic symptoms in EOP, finding that most antipsychotics were efficacious for positive symptoms.^19–21^ Meta-analytical reports from a much smaller cohort of studies showed that antipsychotics reduced negative symptom scores compared with placebo, but no comparisons were statistically significant,^19^ except for clozapine in some studies,^22^ highlighting the need for further research on the treatment of negative symptoms. In the CHR-P field, research on therapeutic advances for children and adolescents has been even more limited, and most studies have focused on other outcomes, such as transition to psychosis.^11,23^

Based on the above, the aim of this study was to (a) evaluate the prevalence of children and adolescents with EOP and at CHR-P who presented with negative symptoms and which factors increased or decreased this prevalence and (b) provide a comprehensive review of the current status and advances in the diagnosis, prognosis and treatment of negative symptoms in children and adolescents with EOP or at CHR-P. Our hypothesis was that negative symptoms would be at least as common in EOP as in adult-onset psychosis and as common in children and adolescents at CHR-P as in children and adolescents with EOP. We further hypothesised that the severity of negative symptoms would be associated with poor outcomes. Finally, we were keen to explore whether evidence for interventions for negative symptoms in both children and adolescents with EOP and children and adolescents at CHR-P was sufficient to recommend specific interventions above others.

## Method

This systematic review and meta-analysis was registered in PROSPERO (CRD42022360925). It was conducted following the guidelines of the Preferred Reporting Items for Systematic Reviews and Meta-Analyses (PRISMA) 2020 statement^24^ (Supplementary eTables 3 and 4) and Meta-analyses of Observational Studies in Epidemiology (MOOSE) checklist (Supplementary eTable 5),^25^ following Enhancing the Quality and Transparency of Health Research (EQUATOR) reporting guidelines.^26^

### Search strategy and selection criteria

We performed a multi-step literature search (keywords in Supplementary eMethods1). First, the PubMed and Web of Science databases (Clarivate Analytics) were searched, incorporating the Web of Science Core Collection, BIOSIS Citation Index, KCI-Korean Journal Database, MEDLINE, Russian Science Citation Index and SciELO Citation Index, as well as the Cochrane Central Register of Reviews and Ovid/PsycInfo databases from inception until 18 August 2022, without language restriction. Second, we searched for data in relevant conference proceedings, including the Schizophrenia International Research Society (SIRS) and Early Intervention in Mental Health international conference (IEPA), as well as in trial registries (clinicaltrials.gov, WHO International Clinical Trials Registry Platform). Following recent guidelines,^27^ search terms were simplified for the search conducted in conference proceedings and trial registries (‘early-onset’, ‘adolescents’ and ‘negative symptoms’). Third, we completed our search by reviewing the references of systematic reviews/meta-analyses retrieved during our search.

Articles identified were screened as abstracts by two researchers working independently (G.S.P., V.S.) and those that were irrelevant were screened out. The full texts of the remaining articles were assessed for eligibility against the inclusion and exclusion criteria, and decisions were made regarding their final inclusion in the systematic review by consensus or mitigation.

Inclusion criteria for the overall review and synthesis were (a) original individual studies, abstracts or conference proceedings, either cross-sectional, longitudinal, randomised clinical trials (RCTs) or other intervention studies, (b) providing relevant information/results on negative symptoms in our populations of interest, (c) conducted in children and adolescents (mean age <18 years, in line with previous reviews on children and adolescents at CHR-P),^10^ (d) conducted in children and adolescents diagnosed with EOP or at CHR-P as per validated instruments and diagnostic criteria (e.g. DSM-any version, ICD-any version and equivalents for EOP, Structured Interview for Psychosis-Risk Syndrome (SIPS), Scale of Psychosis-risk Symptoms (SOPS), Comprehensive Assessment of At-Risk Mental States (CAARMS) and equivalents^28,29^ for CHR-P), (f) published in any language. Exclusion criteria were (a) reviews, editorials or clinical cases, (b) studies reporting on other mental health conditions or with a mean age ≥18 years (since no similar review was found for EOP, studies including EOP individuals >25 years were excluded), (c) studies reporting only negative symptom scores (e.g. Positive and Negative Syndrome Scale scores) without relevant additional results regarding negative symptoms as per established validators, (d) studies without results in children and adolescents with EOP or at CHR-P. Overlap was allowed for the systematic review as long as the key findings were not identical. However, for the meta-analysis, an additional inclusion criterion was that of non-overlapping samples (≤50% overlapping sample) as per our protocol.

### Outcome measures, covariates and data extraction

Researchers (B.P., J.V.S., A.A.) independently extracted data from all included studies into an Excel spreadsheet. Any discrepancies were resolved through consensus or consulting a senior researcher (G.S.P.) when necessary. The variables extracted can be found in Supplementary eMethods2.

### Quality assessment

Quality was assessed using a modified version of the Newcastle–Ottawa Scale (NOS) for cross-sectional and cohort studies. Studies were awarded a maximum of eight points (items can be found in Supplementary eTable 6). Additionally, for RCTs, the Cochrane Risk of Bias Tool (RoB2) was used, and the overall quality was rated as low risk of bias, unclear risk of bias or high risk of bias (Supplementary eMethods 3).

### Qualitative data synthesis

We provided a narrative synthesis of the findings from the included studies. The available evidence was structured into diagnostic factors, prognostic factors and therapeutic factors. Evidence was provided separately for children and adolescents with EOP and at CHR-P.

### Quantitative meta-analysis

Random-effects meta-analytical estimates were computed independently and categorically for children and adolescents with EOP and at CHR-P, including the prevalence of EOP and CHR-P with negative symptoms as per individual study definition. The meta-analyses were conducted using Stata/MP 16.0 with the metaprop package of STATA statistical software (StataCorp) for Mac,^30^ which was developed for pooling proportions in a meta-analysis of multiple studies, and with Comprehensive Meta-Analysis Version 3 for Mac (Biostat, Inc., Englewood, NJ; https://www.meta-analysis.com/?gclid=CjwKCAiAk--dBhABEiwAchIwkcm3gREJ81_iOw3KBdGLG1_Qjf-md9IOeyg_fLmAecxJJwFC6HODAhoCbR0QAvD_BwE). The 95% confidence intervals (CIs) were derived from Wilson score procedures. Publication bias was assessed with the metafunnel function of Stata, which produces funnel plots for assessing small-study reporting bias in meta-analysis, and with Egger's test in the metabias function of Stata. Heterogeneity among study point estimates was assessed using *Q* statistics. The proportion of the total variability in the effect size estimates was evaluated with the *I*^2^ index. Since we expected significant heterogeneity, random-effects models were used.

We conducted sub-analyses and meta-analytical regression analyses to estimate the association between the prevalence of negative symptoms and moderating factors. Sub-analyses included (a) decade of publication (1991–2000, 2001–2010, 2011–2022), (b) continent (Europe, Asia, North America, Africa – owing to availability of data), (c) age (studies including some young adults ≥18 years compared with those with only children and adolescents); (d) design (cross-sectional, longitudinal). Meta-regression analyses evaluated the influence of (a) publication year, (b) percentage of participants with schizophrenia, (c) sample size; (d) mean age, (e) percentage of males, (e) percentage on antipsychotics and (f) quality of the studies (NOS scores) on the results. Statistical significance was considered when *P* < 0.05.

## Results

**Fig. 1:**
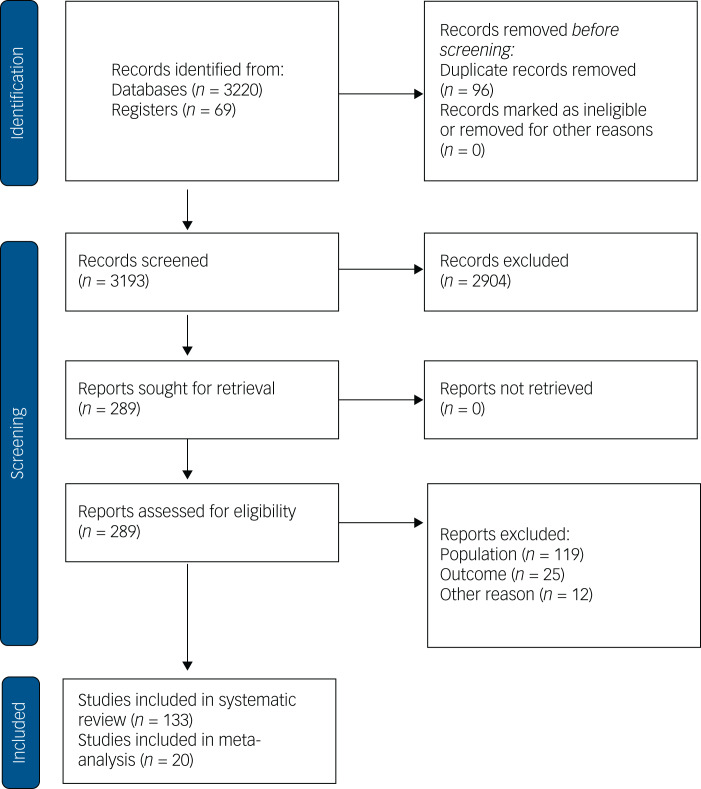
Preferred reporting items for systematic reviews and meta-analyses (PRISMA) flowchart outlining the study selection process.

Our literature search identified 3289 studies; 3193 were screened at title and abstract level and 289 were assessed as full text against inclusion and exclusion criteria. Of those, 133 studies were finally included in the systematic review (Fig. 1): 129 (97%) were written in English and 4 (3%) in other languages; 100 (75.2%) focused on EOP, 29 (21.8%) on CHR-P and 4 (3%) on both. Across all studies, 9055 children and adolescents were included (6776 with EOP, 2138 at CHR-P and 141 without clear designation to one or the other diagnostic group). The sample size of the studies ranged from 10 to 638 (median 45). In total, 68 (51.1%) studies were carried out in Europe, 35 (26.3%) studies in North America, 24 (18.0%) in Asia, 2 (1.5%) in Australia, 1 (0.8%) in South America, 1 (0.8%) in Africa and 2 (1.5%) in more than one continent. Altogether, 127 studies (95.5%) were available as full manuscripts and 6 (4.5%) as abstracts/conference proceedings. Overall, 56 (42.1%) studies were cross-sectional, 52 (39.1%) were longitudinal observational studies, 14 (10.5%) were RCTs, and 11 (8.3%) were other intervention studies. The overall mean age of the participants was 15.5 years (s.d. = 1.6) and 54.1% were males. The mean age of participants with EOP was 15.3 years (s.d. = 1.6) and 56.1% were males. The mean age of participants at CHR-P was 16.1 years (s.d. = 1.0) and 48.6% were males.

### Quality assessment

Overall, the quality of the included studies ranged from 1 to 8, with a median of 5 and a mean of 4.8 (s.d. = 1.4); 4 (28.6%) RCTs were rated as low risk of bias, 4 (28.6%) RCTs were rated as unclear risk of bias and 6 (42.9%) RCTs were rated as high risk of bias (Supplementary eTables 7 and 8). The quality of the studies evaluating children and adolescents with EOP ranged from 1 to 8, with a median of 5 and a mean of 4.7 (s.d. = 1.4) (Supplementary eTable 7). The quality of the studies evaluating children and adolescents at CHR-P ranged from 3 to 8, with a median of 5 and a mean of 5.1 (s.d. = 1.3) (Supplementary eTable 8).

### Systematic review in children and adolescents with EOP

Characteristics of the included studies (*n* = 100) and key findings related to children and adolescents with EOP can be found in Supplementary eTable 7. Findings evaluated at meta-analytical level will be reported separately below.

#### Diagnostic or detection factors

In total, 42 (42.0%) studies focused on diagnostic or detection factors. Findings related to diagnostic categories and subgroups can be found in Supplementary eResults 1, and findings related to neuroanatomical, neuroimaging and other neurobiological non-cognitive findings can be found in Supplementary eResults 2. Findings on clinical factors, functioning and quality of life and cognitive factors are synthesised below.

Altogether, 12 (12.0%) studies focused primarily on comorbidity and clinical factors. Negative symptoms were more severe in children and adolescents with EOP with higher levels of depression (*P* = 0.023).^31^ The prevalence of negative symptoms was associated with enuresis (OR = 1.93, *P* < 0.05) and incontinence during psychosis (OR = 3.35, *P* = 0.005).^32^ No overall differences in negative symptoms between children and adolescents with EOP with and without OCD were found (*P* > 0.05).^33,34^ A positive association was found between negative symptoms and emotional expression (*r* = 0.58, *P* < 0.01), involvement (*r* = 0.54, *P* < 0.05) and recall (*r* = 0.48, *P* < 0.05).^35^ Also, an association was found between more severe negative symptoms and greater emotion regulation impairment (β = 0.31, *P* = 0.02).^36^ Interestingly, children and adolescents with EOP who had never attempted suicide had more negative symptoms during the first episode than those with previous attempts (*P* < 0.05),^37^ and the percentage with a history of suicide attempts was higher among those without persistent negative symptoms (*P* = 0.002).^38^ There was an association between negative symptoms in males and a delayed puberty (*P* = 0.001), which did not appear in females.^39^ A family history of psychosis^40,41^ or family burden^42^ did not seem to have an effect on negative symptoms (*P* > 0.05).

Altogether, 12 (12.0%) studies focused primarily on functioning and quality of life. In EOP, negative symptoms were associated with impairment in premorbid functioning (*P* < 0.01), global functioning (*P* < 0.01), social functioning (*P* < 0.01),^43,44^ role functioning (*P* = 0.003),^44^ daily living skills (*r* = −0.348, *P* < 0.05),^45^ peer relationships (*r* = 0.26, *P* < 0.005),^46^ quality of life (*P* < 0.001)^47^ and general unawareness (*r* = 0.48),^48^ but not with unawareness about psychotic symptoms (*P* > 0.05).^48^ Negative symptoms were increased in children and adolescents with EOP with declining social support compared with those with a stable social support group (*P* < 0.05).^49^

Altogether, 7 (7.0%) studies focused primarily on cognitive factors. More negative symptoms were associated with smell identification deficits (*r* = 0.47, *P* = 0.03),^50^ lower IQ (*r* = 0.41–0.63, *P* < 0.05)^50,51^ and lower performance on some executive function and working memory tasks.^42^ Specifically, negative symptoms were associated with worse speed of processing at baseline (*r* = 0.309, *P* < 0.05) and 6-month follow-up (*r* = 0.184, *P* < 0.05),^52^ more perseverative errors (*r* = 0.31, *P* < 0.05), less phonological fluency (*r* = −0.27, *P* < 0.05), higher number of uncommon responses (*r* = 0.27, *P* < 0.05) and slower response time (*r* = 0.44, *P* = 0.015).^53^ Additionally, specific negative symptoms were associated with specific cognitive domains. This included the association of apathy (β = −0.257, *P* = 0.002) and diminished expression (β = −0.259, *P* = 0.001) with verbal learning; and of diminished expression with speed of processing (β = −0.173, *P* = 0.024).^54^ However, no significant association was found between negative symptoms and attention (*P* < 0.05).^55^

#### Prognostic factors

Altogether, 37 (37%) studies looked at longitudinal prognostic factors. The key factors about the changes in negative symptoms in children and adolescents with EOP and the factors that contribute to these changes are reported below following the same order as above (diagnostic categories and subgroups, clinical factors, functioning and quality of life, neurobiological findings and cognitive factors) including additionally prognostic therapeutic factors.

The trajectory of negative symptoms was variable. One study found worsening of negative symptoms after 6 months^56^ and three others reported consistent and stable negative symptoms,^57–59^ including after as many as 42 years (*P* = 0.935).^59^

Individuals who developed schizophrenia had more negative symptoms than those diagnosed with affective psychosis 1 year after their index admission (*P* = 0.03).^60^ Negative symptoms at 2 years follow-up were more prominent in children and adolescents with EOP with lower baseline general symptoms (*r* = −0.242, *P* = 0.043) and more prominent negative symptoms at baseline (*P* = 0.025).^61^ Duration of untreated psychosis was higher in those with persistent negative symptoms (*P* = 0.022).^38^ Negative symptoms at baseline was the only variable that predicted functional outcome at 2-year follow-up (*P* = 0.010).^62^ Negative symptoms at baseline also predicted lower maximum levels of functioning achieved at 1-year (β = 0.6, *P* = 0.005) and 2-year follow-up (β = 0.5, *P* = 0.003).^51,63^ Greater improvement in negative symptoms correlated with a thinner frontal cortex at baseline (*r* = 0.5, *P* = 0.003).^64^ There was also an association between left frontal cerebrospinal fluid (CSF) volume increase during follow-up (*r* = 0.58; *P* = 0.003) and left parietal CSF increase (*r* = 0.45; *P* = 0.03) and negative symptoms.^65^ Interestingly, negative symptoms at baseline predicted an improvement in executive performance after 2 years (β = 4.688, *P* = 0.008).^66^ Finally, negative symptoms at admission were predictors of poor treatment efficacy in EOP (OR = 0.945, *P* = 0.009).^67^ In fact, negative symptoms were significantly associated with multiple treatment failures (HR = 1.62, *P* = 0.02).^6^

#### Therapeutic factors

Altogether, 37 (37%) studies looked directly or indirectly at therapeutic factors and response to interventions. Participants were not selected based on the presence of negative symptoms. The key advances in pharmacological interventions in RCTs, pharmacological interventions in other clinical trials, psychosocial interventions in RCTs and psychosocial interventions in other clinical trials are detailed below.

Evidence in EOP coming from RCTs was limited for pharmacological interventions. Clozapine decreased negative symptoms in treatment-resistant EOP compared with haloperidol in a double-blind RCT (*P* = 0.002).^68^ Clozapine was also more efficacious than olanzapine in reducing negative symptoms in treatment-resistant EOP after 8 weeks (*P* = 0.04; *d* = 0.89)^69^ and 12 weeks (*P* = 0.02, *d* = 0.92)^70^ in another RCT. Treatment with lurasidone compared with placebo was associated with greater improvement in negative symptoms for children and adolescents with previously treated EOP (*P* = 0.017; s.m.d. = 0.32) but not in treatment-naive children and adolescents with EOP.^71^ With regard to evidence from other intervention studies, negative symptoms improved after 20 days (*P* < 0.001)^72^ and 88 weeks^73^ in an open-label trial with quetiapine. One study in children and adolescents with EOP found that negative symptoms responded better to aripiprazole than positive symptoms (*P* = 0.028).^74^ In a randomised open-label study comparing olanzapine with risperidone, negative symptoms improved more with olanzapine after 8 weeks (*P* < 0.01)^75^ and after 1 year,^76^ and a ≥50% reduction in negative symptoms was achieved more frequently with olanzapine (41.7%) than with risperidone (7.7%) (*P* = 0.047) (note that this difference disappeared following Bonferroni correction).^77^ However, efficacy was similar for risperidone (14% decline in negative symptoms), olanzapine (17.7% decline) and haloperidol (19.2% decline) in another study.^78^ No differences in negative symptom reduction were found between quetiapine and olanzapine (*P* > 0.05).^79^ Similarly, no difference in efficacy for negative symptoms emerged between paliperidone and aripiprazole after 2 months (*P* = 0.535) or 6 months (*P* = 0.696).^80^

Evidence from RCTs was also limited for psychosocial interventions. In one RCT a psychoeducation group for children and adolescents with EOP and their parents showed a greater reduction in negative symptoms than the non-structured group (*r* = 0.41).^81^ However, the improvement did not persist after 2 years.^82^ There was, though, an association between improvements in executive function and a reduction in negative symptoms (*P* < 0.05) in the psychoeducation group.^83^ With regard to evidence from other intervention studies, participants attending a programme of residential out-patient care following discharge from a clinic showed a significantly greater decrease in negative symptoms than the control group (*P* = 0.002).^84^ No differences in negative symptom improvement were found between cognitive–behavioural therapy (CBT) added to treatment as usual and treatment as usual only at the end of the intervention (*P* = 0.317), as well as at 9-month (*P* = 0.169) and 18-month follow-up (*P* = 0.086).^85^

### Systematic review in children and adolescents at CHR-P

Characteristics of the included studies (*n* = 33) and key findings related to CHR-P can be found in Supplementary eTable 8. Findings evaluated at meta-analytical level will be reported separately below.

#### Diagnostic or detection factors

Altogether, 13 (39.4%) studies focused primarily on diagnostic or detection factors. The key diagnostic and detection factors in CHR-P are reported below following the same order as above (diagnostic categories and subgroups, clinical factors, functioning and quality of life, neurobiological findings and cognitive factors).

More severe negative symptoms were associated with greater illness severity (*r* = −039, *P* > 0.001),^86^ poorer current global functioning (*r* = −0.26; *P* = 0.015),^87^ social functioning (*r* = 0.38–0.47, *P* ≤ 0.001)^87,88^ and role functioning (*r* = −0.25; *P* = 0.025),^87^ lower current functioning (*r* = −0.17; *P* = 0.031), lower lowest functioning in the past year (*r* = −0.20; *P* = 0.014) and lower highest functioning in the past year (*r* = −0.19; *P* = 0.022).^86^ A correlation between negative symptoms and depressive symptoms was also observed (*r* = 0.380–533, *P* < 0.01),^89,90^ particularly anhedonia (*P* < 0.001).^91^ No correlations between negative symptoms and attachment (*P* > 0.05)^92^ were found. No differences in negative symptoms were found between children and adolescents at CHR-P with attenuated negative/disorganised symptoms only, those with attenuated positive symptoms and those with schizophrenia-like psychosis (*P* > 0.05).^93^ More individuals with negative symptoms were found among children and adolescents at CHR-P with major depressive disorder than without major depressive disorder (90.3% *v*. 68.2%, *P* = 0.021).^89^ One study reported that 70% of children and adolescents at CHR-P experienced a decrease in the ability to start/maintain social relationships, 80% experienced poor work and school performance and 55% experienced social withdrawal.^94^

Negative symptoms were more severe in children and adolescents at CHR-P with 22q11 deletion syndrome than without the syndrome (*P* = 0.0081).^95^ From a neuroimaging perspective, larger left amygdala volumes were associated with negative symptoms in females (*P* = 0.020) but not in males.^96^ Negative symptoms were associated with worse processing speed (*r* = −0.31, *P* = 0.014) and verbal performance (*r* = −0.37, *P* = 0.03)^97^ as well as the total speed of timed activities (*P* = 0.038).^98^ Moreover, different negative symptom dimensions were associated with difficulties in metacognition (i.e. cognition related to cognitive impairments) (*P* < 0.001).^99^ However, no significant associations emerged between negative symptoms and neurocognitive measures in another study (*P* > 0.05).^88^

#### Prognostic and therapeutic factors

Altogether, 20 (60.6%) studies focused on longitudinal prognostic or therapeutic factors. The key factors regarding changes in negative symptoms in children and adolescents at CHR-P and the factors that contribute to these changes are reported below following the same order as above (diagnostic categories and subgroups, clinical factors, functioning and quality of life, neurobiological findings and cognitive factors, therapeutic factors). Participants in clinical trials were not selected based on the presence of negative symptoms.

Males had more severe negative symptoms than females at 6-month and 12-month follow-up (*P* < 0.05).^100^ Negative symptoms at baseline did not predict transition to psychosis (*P* = 0.76) in one study (*n* = 71, 1-year follow-up),^101^ whereas they did in another (*n* = 153, 7-year follow-up) (AUC = 0.74, *P* < 0.01).^102^ In fact, conversion was best predicted by negative symptoms compared with other clinical variables in children and adolescents at CHR-P (*P* = 0.006, *d* = 0.46),^103^ and 100% of children and adolescents at CHR-P who transitioned to psychosis had negative symptoms in a further study.^13^ Additionally, positive remarks by family members were associated with decreased negative symptoms (*P* < 0.05).^104^

In one RCT, omega-3 fatty acid treatment was associated with significantly lower negative symptom scores at 12 weeks (*P* < 0.05), 6 months (*P* < 0.05) and 12 months (*r* = 0.52, *P* < 0.05) compared with placebo.^105^ More severe baseline negative symptoms were associated with treatment response in the omega-3 supplemented group compared with the placebo group (*d* = 0.7).^106^ However, no significant differences in negative symptoms between the groups receiving CBT and risperidone, CBT and placebo, supportive therapy and placebo, and monitoring only were found in another study (*P* > 0.05).^107^ Family-focused treatment was also not associated with an improvement in negative symptoms (*P* > 0.05). Children and adolescents at CHR-P who were on antipsychotics showed greater improvement in negative symptoms than those not on antipsychotics (*P* = 0.03) in a family-focused treatment trial.^108^

### Meta-analyses on the prevalence of negative symptoms

Twenty studies and 1799 individuals were included in the meta-analysis: 1457 children and adolescents with EOP (mean age 15.5 years (s.d. = 1.2), 52.1% males) and 342 children and adolescents at CHR-P (mean age 15.2 years (s.d. = 0.9), 52.6% males). Note that this is 15% of the included studies: the rest were not meta-analysed as they evaluated negative symptoms continuously (i.e. their severity) or were overlapping with these studies. Altogether, 66.0% (95% CI 53.6–78.5%) of the total sample had negative symptoms (*k* = 20 studies, *n* = 1799). Heterogeneity (*I*^2^) across the included studies was statistically significant (*I*^2^ = 98.0%, *P* < 0.001). Publication bias was not detected in the funnel plot (Supplementary eFig. 1) or Egger's test (*P* = 0.395) (Supplementary eTable 9). Notably, no overlap was found in any of the studies included in the meta-analysis.

**Fig. 2:**
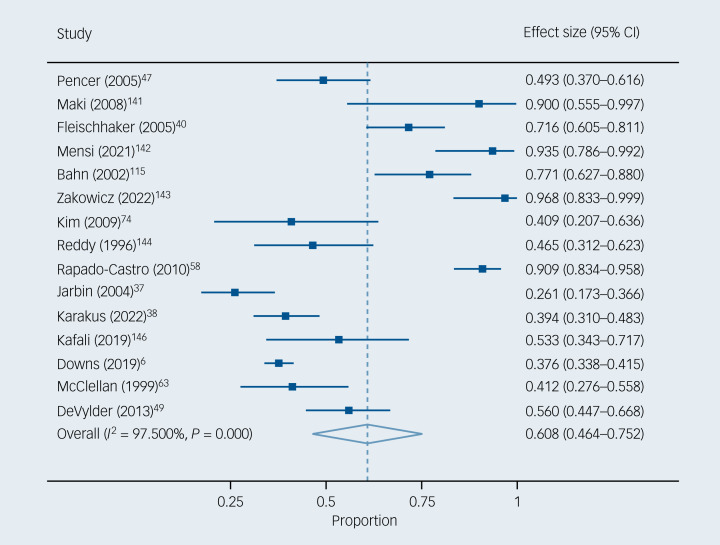
Negative symptom prevalence in children and adolescents with early-onset psychosis.

**Fig. 3:**
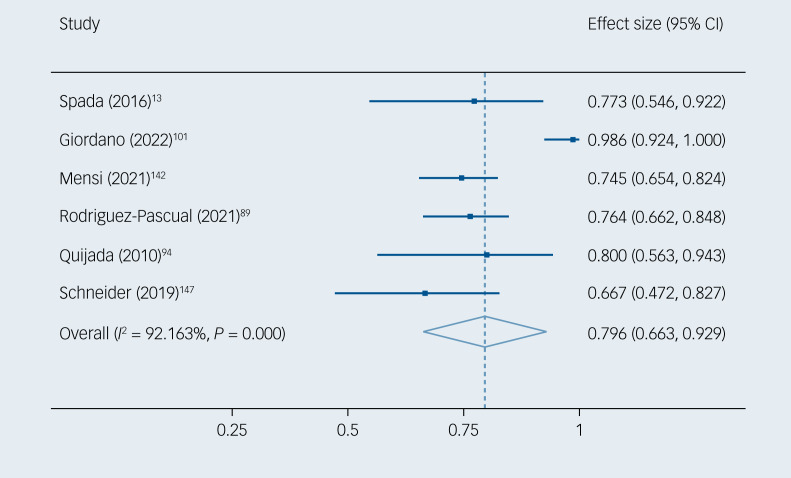
Negative symptom prevalence in children and adolescents at clinical high-risk for psychosis.

Of the children and adolescents with EOP, 60.8% (95% CI 46.4–75.2%) had negative symptoms (*k* = 15, *n* = 1457) (Fig. 2). Heterogeneity across the included studies was statistically significant (*I*^2^ = 97.5%, *P* < 0.001). Publication bias was not detected in the funnel plot (Supplementary eFig. 2) or Egger's test (*P* = 0.578) (Supplementary eTable 9). Of the children and adolescents at CHR-P, 79.6% (95% CI 66.3–92.9%) had negative symptoms (*k* = 6, *n* = 342) (Fig. 3). Heterogeneity across the included studies was statistically significant (*I*^2^ = 92.2%, *P* < 0.001). Publication bias was not detected in the funnel plot (Supplementary eFig. 3) or Egger's test (*P* = 0.057) (Supplementary eTable 9).

### Sub-analyses and meta-regression analyses

In the sub-analyses, the decade of publication seemed to moderate the prevalence of negative symptoms (*Q* = 10.427, *P* = 0.005). Studies published in 2011–2022 detected negative symptoms in 70.3% (95% CI 56.6–81.1%) of children and adolescents with EOP or at CHR–P, whereas studies published in 2001–2020 detected negative symptoms in 66.0% (95% CI 43.2%–83.2%) and studies published in 1991–2000 detected negative symptoms in 43.6% (95% CI 34.0%–53.8%).

The continent of publication also moderated the prevalence of negative symptoms (*Q* = 9.145, *P* = 0.027). Studies published in Europe (72.6%, 95% CI 59.2–82.9%) and Asia (60.9%, 95% CI 24.9–88%) found a higher prevalence of negative symptoms than those published in North America (49.7%, 95% CI 41.6–57.8%) or Africa (46.5%, 95% CI 32.3–61.3%).

No differences were found between studies including some individuals ≥18 years compared with those studies including only individuals <18 years (*Q* = 0.026, *P* = 0.871) or between cross-sectional studies and longitudinal studies (*Q* = 0.020, *P* = 0.889) (Table 1).

**Table 1:** Subgroup analyses

Group, subgroup	Studies, *k*	Participants, *n*	Meta-analysis		*z*-score	*P*	Heterogeneity		Within subgroup heterogeneity	
			Proportion	95% CI			*I*^2^	*P*	*Q*	*P*
Decade	20	1799	0.660	0.536 to 0.785	2.969	0.003	98.000	<0.001	10.427	0.005
1991–2000	2	94	0.436	0.340 to 0.538	−1.229	0.219	0.0	0.604		
2001–2010	7	389	0.660	0.432 to 0.832	1.384	0.166	92.588	<0.001		
2011–2022	11	1316	0.703	0.566 to 0.811	2.837	0.005	92.312	<0.001		
Continent	20	1799	0.660	0.536 to 0.785	2.969	0.003	98.000	<0.001	9.145	0.027
Europe	14	1482	0.726	0.592 to 0.829	3.165	0.002	94.376	<0.001		
Asia	2	70	0.609	0.249 to 0.880	0.563	0.573	87.755	0.004		
North America	3	204	0.497	0.416 to 0.578	−0.073	0.942	27.919	0.250		
Africa	1	43	0.465	−0.323 to 0.613	-0.457	0.648	0.000	1.000		
Design	18	1677	0.655	0.564 to 0.736	3.268	0.001	92.312	<0.001	0.026	0.871
Age range 0–18 years	13	1480	0.662	0.537 to 0.768	2.506	0.012	93.775	<0.001		
Age range includes >18-year-olds	5	197	0.647	0.510 to 0.765	2.104	0.035	68.837	0.012		
Design	20	1799	0.660	0.536 to 0.785	2.969	0.003	98.000	<0.001	0.020	0.889
Cross-sectional	6	314	0.651	0.528 to 0.757	2.384	0.017	93.563	0.001		
Longitudinal	14	1485	0.663	0.531 to 0.774	2.406	0.016	75.713	<0.001		

In the meta-regression analyses, neither publication year, percentage with schizophrenia, sample size, mean age, percentage of males, percentage on antipsychotics nor NOS scores were significantly associated with the prevalence of negative symptoms (all *P* > 0.05) (Table 2).

**Table 2:** Meta-regression analyses

Variable	Studies, *k*	β coefficient	s.e.	95% CI	z-score	*P*
Year of publication	20	0.0511	0.0291	−0.0059 to 0.1082	1.76	0.0791
Percentage of patients with schizophrenia	11	0.0004	0.0109	−0.0210 to 0.0217	0.03	0.9737
Sample size	20	−0.0022	0.0016	−0.0053 to 0.0009	−1.41	0.1572
Mean age	17	−0.1314	0.2488	−0.6190 to 0.3562	−0.53	0.5974
Percentage of males	18	0.0187	0.0219	−0.0242 to 0.0616	0.85	0.3934
Percentage on antipsychotics	7	−0.0021	0.0080	−0.0177 to 0.0136	−0.26	0.7966
Quality of the study	20	−0.0915	0.1386	−0.3631 to 0.1801	−0.66	0.5092

## Discussion

To the best of our knowledge, this is the first systematic review to provide a comprehensive evaluation of the evidence regarding negative symptoms and diagnostic, prognostic and therapeutic factors in children and adolescents with EOP or at CHR-P. Additionally, this is the first quantitative meta-analysis on this topic. We systematically reviewed 133 studies evaluating 6776 children and adolescents with EOP and 2138 children and adolescents at CHR-P, and conducted the first meta-analysis on the prevalence of negative symptoms in children and adolescents in 20 studies involving 1799 individuals (1457 with EOP and 342 at CHR-P) for those studies evaluating the presence/absence of negative symptoms and independent of whether they also reported on the dimensional severity of negative symptoms. The prevalence of negative symptoms in children and adolescents with EOP was 61% and the prevalence in those at CHR-P was 80%. In general, negative symptoms were associated with poorer clinical, functional, neurobiological, cognitive and intervention outcomes in both children and adolescents with EOP and children and adolescents at CHR-P, as detailed in the included studies and the systematic review above. Various interventions in heterogeneous populations have been piloted with variable results that require further study replication. Overall, these findings suggest that negative symptoms are frequent and clinically relevant both in children and adolescents with EOP and in those at CHR-P.

### Negative symptoms in EOP

One of our main findings is that over 60% of children and adolescents with EOP experience negative symptoms when these are evaluated. Negative symptoms seem to appear in about 30–50% of individuals with an adult-onset first episode of psychosis.^109,110^ The prevalence observed was thus 20–100% higher in children and adolescents with EOP than in adults with a first episode of psychosis. There are different hypotheses or explanations for these results. On the one hand, it may be that this higher prevalence is due to other characteristics of children and adolescents with EOP, which may in turn be associated with poor prognosis. For instance, children and adolescents with EOP present with more neurodevelopmental difficulties,^111^ poorer premorbid adjustment,^7^ more cognitive impairment and higher impulsivity^112^ than individuals with adult-onset psychosis. Alternatively, it may be that the participants were particularly enriched in risk factors.^113^ Of note, individual studies found that children and adolescents with EOP showed negative symptoms more frequently, and that an earlier age at onset was associated with a higher number of (and more severe) negative symptoms.^114,115^ In any case, the assessment and management of negative symptoms in children and adolescents with EOP should be prioritised. Psychiatrists and other mental health professionals should actively and comprehensively evaluate negative symptoms in young people. This includes child and adolescent mental health service clinicians treating children and adolescents with EOP who present to their clinic for the first time, and adult clinicians when these patients transition to their services. On a positive note, our sub-analyses suggest that in the past two decades the identification of negative symptoms has improved globally, at least in research studies. Nevertheless, a potential reason for health professionals not focusing on negative symptoms clinically may be rooted in the fact that high-level evidence for specific treatments for negative symptoms is lacking.^116^ In adults with schizophrenia, at least, antidepressants^117,118^ and aerobic exercise^119^ have been shown to improve negative symptoms, but no such trial data exist for children and adolescents with EOP.

### Negative symptoms in CHR-P

Another particularly relevant finding is that negative symptoms appeared in almost 80% of children and adolescents at CHR-P and that this prevalence was higher than for children and adolescents with EOP; importantly, these findings held true at a meta-analytical level. It seems that negative symptoms are observed in the context of emerging attenuated positive symptoms during the prodromal period before the first episode of psychosis. Previous evidence suggests that negative symptoms may be the most common first symptoms of schizophrenia,^3^ potentially appearing 1 year before the emergence of attenuated positive symptoms.^120^ This sequence of events has led some researchers to suggest that individuals with negative symptoms should be included as a new clinical risk group for developing psychosis.^13,121^ However, one prospective cohort study found that the conversion to psychosis in the group with negative symptoms only was about 5% at 5 years,^121^ indicating that negative symptoms alone have limited positive predictive validity. The implication is that screening instruments^122^ and CHR-P services^123^ should continue identifying ‘high risk’ individuals on the basis of attenuated positive clinical symptoms.^122^ To note, clinical services to prevent psychosis do typically focus on (attenuated) positive symptoms^123^ in their initial assessment to identify children and adolescents at CHR-P. An alternative explanation for the higher prevalence of negative symptoms in CHR-P could simply be that the instruments used to detect negative symptoms in children and adolescents are more sensitive. To note, negative symptoms were found to be less severe in children and adolescents with EOP than in those at CHR-P fulfilling DSM-5 criteria for attenuated psychosis syndrome (*P* < 0.001).^124^ However, the power of our analysis was lower for children and adolescents at CHR-P than for those with EOP, with just six independent studies fulfilling our CHR-P inclusion criteria.

### Diagnostic or detection factors

From a diagnostic perspective, we have extensively reviewed clinico-epidemiological, neurobiological and neurocognitive risk factors that increase the likelihood of experiencing negative symptoms or their severity. In our meta-regression analysis, sample prevalence of schizophrenia was not associated with an increased prevalence of negative symptoms. Individual studies did find a greater severity of negative symptoms in the schizophrenia subgroup.^125^ This finding suggests that negative symptoms do not only appear in schizophrenia and should be evaluated and monitored in children and adolescents in the early stages of psychosis regardless of their diagnosis or presentation. That said, there may be some individuals in whom the therapeutic intensity of pharmacological interventions, particularly antipsychotic treatment, could be minimised and psychosocial interventions could be offered instead. For instance, young people with brief psychotic disorders seem to present less severe negative symptoms, not only compared with schizophrenia (*P* = 0.006)^126^ but also compared with psychosis ‘not otherwise specified’ (*P* = 0.02).^127^ This finding is in line with previous suggestions of offering psychosocial interventions without antipsychotic medication to individuals with brief psychotic episodes^128^ or with a shorter duration of untreated psychosis,^129^ which is in turn associated with less severe negative symptoms.^130^

Risk factors may also have an effect on the presentation of negative symptoms. For instance, children and adolescents with EOP and obesity seemed to present with less severe negative symptoms (*P* = 0.003).^131^ It is therefore important to advance knowledge on the implementation of precision psychiatry to be able to offer state-of-the-art interventions that are personalised and needs-based.^132^ To do that, it is vital to provide mental health professionals with adequate competence and skills training to identify and manage relevant psychopathological and functional disabilities, including those related to negative symptoms.^133^ Notably, the detection of negative symptoms seems to be improving in recent decades, the prevalence of negative symptoms having increased from 44% in 1991–2000 to 70.3% in 2011–2022, suggesting that some of these competencies and skills have been achieved by professionals. One of the challenges in this field, regarding the acquisition of some of these competencies, is the distinction between depressive symptoms and negative symptoms, since there is a correlation between depressive symptoms and negative symptoms and since there may be some overlap, including in individuals with non-affective psychosis.^18,86,89,90^ A systematic review identified that depressed mood, hopelessness and suicidality had greater specificity for depression in people with schizophrenia, whereas alogia, affective blunting and social withdrawal were more characteristic of negative symptoms.^134^ These distinctions are not always easy for clinicians to make, but as a recent network analysis in adults with schizophrenia and predominant negative symptoms showed, negative symptoms appear to be an independent symptom cluster that can be delineated from depressive symptoms in the network.^135^ Psychometric instruments or digital tools that clearly differentiate these symptoms are required since the presence and severity of negative symptoms may overlap or covary with the severity of other symptoms (e.g. with depressive symptoms but also with positive symptoms or anxiety symptoms) and with functional impairment. Training in the use of these instruments is therefore also important. Finally, the development of a core outcome set for observational and clinical studies in EOP and CHR-P individuals (as per the Core Outcome Measures in Effectiveness Trials: www.comet-initiative.org) that does not rely only on behaviour could reduce heterogeneity and measurement variation and improve the accuracy of the detection of negative symptoms.

### Prognostic factors

From a prognostic perspective, this review provided valuable information on diagnostic stability, course and outcomes as well as on the factors that are associated with increased negative symptoms during longitudinal follow-up. Overall, negative symptoms were characterised by consistency and stability.^57,58^ Interestingly, meta-analytical evidence showed that negative symptoms improved at 12-month follow-up (*g* = 0.496) but not at 24 months or ≥36 months compared with baseline (*P* > 0.05) in CHR-P individuals.^136^ This result suggests that negative symptoms need to be monitored during the follow-up period, even if they initially improve. Some children and adolescents with EOP or at CHR-P present with clinical risk factors for poor outcomes (e.g. prominent negative symptoms at baseline^61^ or long duration of untreated psychosis)^38^ or neurobiological risk factors for poor outcomes (e.g. frontal cortical thinness, changes in the cerebrospinal fluid)^64^. They may require additional clinical attention since their negative symptoms may deteriorate.

### Therapeutic factors

Finally, from a therapeutic perspective, we conclude that research on preventive treatments for children and adolescents with EOP or at CHR-P has limited evidence compared with research in adults.^137^ In the reviewed studies in children and adolescents with EOP, clozapine was the only medication that showed in RCTs superiority against other antipsychotic medications.^68,69^ However, since meta-analytical evidence shows that clozapine is associated with significant cardiometabolic, cardiac, haematological and neurological adverse effects in children and adolescents,^138,139^ clozapine needs to be reserved for treatment-resistant cases in which they have been researched (e.g. after two previous antipsychotics have failed). Other medications, such as aripiprazole, for which a study found a better response in children and adolescents with EOP with negative symptoms than with positive symptoms,^74^ may be prescribed first. Other second-generation antipsychotics (e.g. lurasidone^71^ and quetiapine^72,73^) have shown some benefits, but the comparisons do not clearly benefit any of them (apart from clozapine) over the others. The study of other medication groups is recommended, particularly antidepressants, which have shown to be overall effective – although with small effect size – for negative symptoms in adults.^117,118^ There is also insufficient evidence to recommend any specific psychosocial intervention in children and adolescents with EOP or at CHR-P over the others. Of note, early intervention services (typically offered to adolescents and young adults with EOP or at CHR-P) have shown a reduction in negative symptom severity after 6–24 months^140^ compared with treatment as usual, supporting the need for funding and use of early intervention services. To advance knowledge in the field, future research should evaluate changes in negative symptoms as their primary outcome, recruiting and selecting children and adolescents in whom negative symptoms are predominant.

### Limitations and strengths

This study has several limitations that must be taken into consideration when interpreting our results. First, the sample sizes and the number of articles were limited for some of the evaluated outcomes. Importantly, only 20 independent samples provided independently meta-analysable data on the presence of negative symptoms, because most studies only reported negative symptoms as a continuous outcome. Second, participants included in the studies were heterogeneous and not selected on the basis of the presence of negative symptoms; the studies were also heterogeneous in their design, methodology and quality, which was low in some of the included studies. Third, the threshold used to consider that negative symptoms were present varied, and some studies did not specify how they measured or defined the presence of negative symptoms. Also, currently used instruments typically rely on behaviour that may have been reported and not always observed, whereas subjective experiences may be insufficiently assessed. Fourth, the mean age of participants in the included studies ranged from 10–17.9 years, which was highly variable. Differences in terms of neurodevelopment and subsequent expression of negative symptoms could exist. We have mitigated against this problem with our sensitivity analyses, but some of these analyses may have been underpowered. Finally, and relatedly, as anticipated in our protocol, the amount of evidence was limited for some outcomes and did not allow us to carry out additional meta-analyses of longitudinal data.

This study also has several strengths. Among them is the fact that this is the ‘first in field’ and most comprehensive systematic review with meta-analytical evidence to date focusing on the prevalence of negative symptoms in children and adolescents with EOP and at CHR-P. Our database for the systematic review was large and globally representative, including 133 individual studies. We used rigorous methods and carefully reported study quality, while providing sensitivity analyses, heterogeneity analyses and publication bias assessments. This approach has allowed us to provide state-of-the-art evidence on the current state in the field but also on the challenges and gaps that future studies should address.

In summary, our findings suggest that negative symptoms are at least as common in children and adolescents with EOP as in adult-onset psychosis, that they appear frequently during the prodromal period in children and adolescents at CHR-P and that they are associated with poor clinical, functional and intervention outcomes in both groups. They highlight the need for further interventional research, so that children and adolescents can receive evidence-based treatments for negative symptoms aimed at improving outcomes.

## Data Availability

This paper reports meta-analytic data based on original published studies. The corresponding author, G.S.P., may be contacted for further information.

## Supplementary material

For supplementary material accompanying this paper visit https://doi.org/10.1192/bjp.2022.203. click here to view supplementary material
