# Supplementary material for: Negative symptoms in children and adolescents with early-onset psychosis and at clinical high-risk for psychosis: systematic review and meta-analysis
Source: Br J Psychiatry. 2023 Jul;223(1):282–94. doi: 10.1192/bjp.2022.203 (PMC10331322; doi:10.1192/bjp.2022.203)
Supplement: Supplementary file 1 [file S0007125022002033sup001.docx]

SUPPLEMENTARY MATERIAL

**eTable1.** Diagnostic Criteria for Ultra-high risk clinical High-Risk for Psychosis (CHR-P) criteria according to SIPS/SOPS.…………...…….page 2

**eTable2.** Diagnostic criteria for Basic Symptoms…….……………………………………………………………………………….………….page 3

**eTable3.** PRISMA 2020 Statement and Checklist…...………..…………..…………..…………..…………..…………..…………..………..page 4-7

**eTable4.** PRISMA 2020 Abstract Checklist……………..………....…………..…………..…………..…………..…………..….….…...….…..page 8

**eTable5.** Moose Checklist...………..…………..…………..…………..…………..…………..…………..…………..…………….………..page 9-11

**eTable6.** Risk of bias (quality) assessment using modified Newcastle Ottawa Scale for cross-sectional and cohort studies……..…….…..….page 12

**eTable7.** Characteristics of included studies (samples with EOP individuals only)……..………………..…………..………… …....……..page 13-26

**eTable8.** Characteristics of included studies (samples with CHR-P individuals)..…… ……………………..………………….…………..page 27-30

**eTable9.** Publication bias: Egger’s test …………..…………..…………..…………..…………..…………..…………..…………..…….…....page 31

**eFig1.** Publication bias: funnel plot EOP and CHR-P…..………..…………..…………………………………..…………..………………….page 32

**eFig2.** Publication bias: funnel plot EOP …………..………..……..…………………………………..…………..…………………………….page 33

**eFig3.** Publication bias: funnel plot CHR-P …………..………..……..…………………………………..…………..………………………….page 34

**eMethods 1.** Search terms……………………….………..…………..…………………………………..…………..………………………….page 35

**eMethods 2.** Summary of included variables…………………………………………………………………………………………………….page 36

**eMethods3.** Risk of bias (quality) assessment with Cochrane Risk of Bias tool for randomized clinical trials…………………………………page 37

**eResults1.** Diagnostic categories and subgroups and negative symptoms in EOP………………….……………………………….…………...page 38

**eResults2:** Neuroanatomical, neuroimaging and other neurobiological non-cognitive findings and negative symptoms in EOP...……...........page 39

This supplementary material has been provided by the authors to give readers additional information about their work.

**eTable1. Diagnostic Criteria for Ultra-high risk clinical High-Risk for Psychosis (CHR-P) criteria according to SIPS/SOPS^1,2^.**

| CHR-P | Attenuated psychotic symptoms | Brief limited intermittent psychotic symptoms | Genetic risk and deterioration syndrome |
| --- | --- | --- | --- |
| Symptoms | SIPS positive symptom scales: (P1) unusual thought content, (P2) suspiciousness, (P3) grandiose ideas, (P4) perceptual abnormalities, and (P5) disorganized communication, with at least 1 of these symptoms rated 3, 4, or 5, indicating clinically significant disturbance below a psychotic level of intensity. | SIPS positive symptom scales: (P1) unusual thought content, (P2) suspiciousness, (P3) grandiose ideas, (P4) perceptual abnormalities, and (P5) disorganized communication, with at least 1 of these symptoms rated, 6 indicating clinically severe disturbance with psychotic features. | The patient meets criteria for Schizotypal Personality Disorder OR has a first-degree relative with a psychotic disorder. |
| Frequency | Symptoms ever been present at an average frequency of at least once per week over a month. | Symptoms ever been present at least several minutes per day, at least one month. |  |
| Onset | Symptoms should have begun within the past year OR currently rate one or more scale points higher compared to 12 months before. Symptoms that occurred over the past month only are rated. | Symptoms should have reached a psychotic level of intensity in the previous 3 months. |  |
| Level of functioning | No social/occupational dysfunction requirement. | No social/occupational dysfunction requirement. | 30% drop in GAF score over the last month as compared to 12 months before. |
| Duration |  | Up to 3 months. |  |
| SIPS: Structured Interview of Psychosis-risk Syndromes; SOPS: Scale of Prodromal Symptoms. | | | |

**eTable2. Diagnostic criteria for Basic Symptoms^3,4^.**

| Basic Symptoms | COPER | COGDIS |
| --- | --- | --- |
| BS are subtle, subjectively experienced disturbances in mental processes including thinking, attention, perception, speech, stress tolerance, and affect. | Presence of at least any one of the following ten basic symptoms, with at least weekly occurrence within the last three months and first occurrence at least 12 months ago:   - Thought interference. - Thought perseveration. - Thought pressure. - Thought blockages. - Disturbance of receptive speech. - Decreased ability to discriminate between ideas and perception, fantasy and true memories. - Unstable ideas of reference. - Derealisation. - Visual perception disturbances. - Acoustic perception disturbances. | Presence of at least any two of the following nine basic symptoms, with at least weekly occurrence within the last three months:   - Inability to divide attention. - Thought interference. - Thought pressure. - Thought blockages. - Disturbance of receptive speech. - Disturbance of expressive speech. - Unstable ideas of reference. - Disturbances of abstract thinking. - Captivation of attention by details of the visual field. |
| BS are regarded as an immediate symptomatic expression of the neurobiological processes underlying psychosis and the earliest form of self‐experienced symptoms. |  |  |
| Two criteria for the identification of basic symptoms:   - Cognitive‐Perceptive Basic Symptoms (COPER). - Cognitive Disturbances (COGDIS). |  |  |

**eTable3. PRISMA 2020 Statement and Checklist.**

| **Section and Topic** | **Item #** | **Checklist item** | **Location where item is reported** |
| --- | --- | --- | --- |
| **TITLE** | | |  |
| Title | 1 | Identify the report as a systematic review. | Title page |
| **ABSTRACT** | | |  |
| Abstract | 2 | See the PRISMA 2020 for Abstracts checklist. | Abstract page |
| **INTRODUCTION** | | |  |
| Rationale | 3 | Describe the rationale for the review in the context of existing knowledge. | Introduction |
| Objectives | 4 | Provide an explicit statement of the objective(s) or question(s) the review addresses. | Introduction |
| **METHODS** | | |  |
| Eligibility criteria | 5 | Specify the inclusion and exclusion criteria for the review and how studies were grouped for the syntheses. | Methods |
| Information sources | 6 | Specify all databases, registers, websites, organisations, reference lists and other sources searched or consulted to identify studies. Specify the date when each source was last searched or consulted. | Methods |
| Search strategy | 7 | Present the full search strategies for all databases, registers and websites, including any filters and limits used. | eMethods 1 |
| Selection process | 8 | Specify the methods used to decide whether a study met the inclusion criteria of the review, including how many reviewers screened each record and each report retrieved, whether they worked independently, and if applicable, details of automation tools used in the process. | Methods |
| Data collection process | 9 | Specify the methods used to collect data from reports, including how many reviewers collected data from each report, whether they worked independently, any processes for obtaining or confirming data from study investigators, and if applicable, details of automation tools used in the process. | Methods |
| Data items | 10a | List and define all outcomes for which data were sought. Specify whether all results that were compatible with each outcome domain in each study were sought (e.g. for all measures, time points, analyses), and if not, the methods used to decide which results to collect. | eMethods 2 |
|  | 10b | List and define all other variables for which data were sought (e.g. participant and intervention characteristics, funding sources). Describe any assumptions made about any missing or unclear information. | eMethods 2 |
| Study risk of bias assessment | 11 | Specify the methods used to assess risk of bias in the included studies, including details of the tool(s) used, how many reviewers assessed each study and whether they worked independently, and if applicable, details of automation tools used in the process. | eMethods 3 |
| Effect measures | 12 | Specify for each outcome the effect measure(s) (e.g. risk ratio, mean difference) used in the synthesis or presentation of results. | Methods |
| Synthesis methods | 13a | Describe the processes used to decide which studies were eligible for each synthesis (e.g. tabulating the study intervention characteristics and comparing against the planned groups for each synthesis (item #5)). | Methods |
|  | 13b | Describe any methods required to prepare the data for presentation or synthesis, such as handling of missing summary statistics, or data conversions. | Methods |
|  | 13c | Describe any methods used to tabulate or visually display results of individual studies and syntheses. | Methods |
|  | 13d | Describe any methods used to synthesize results and provide a rationale for the choice(s). If meta-analysis was performed, describe the model(s), method(s) to identify the presence and extent of statistical heterogeneity, and software package(s) used. | Methods |
|  | 13e | Describe any methods used to explore possible causes of heterogeneity among study results (e.g. subgroup analysis, meta-regression). | Methods |
|  | 13f | Describe any sensitivity analyses conducted to assess robustness of the synthesized results. | Methods |
| Reporting bias assessment | 14 | Describe any methods used to assess risk of bias due to missing results in a synthesis (arising from reporting biases). | eMethods 3 |
| Certainty assessment | 15 | Describe any methods used to assess certainty (or confidence) in the body of evidence for an outcome. | Methods |
| **RESULTS** | | |  |
| Study selection | 16a | Describe the results of the search and selection process, from the number of records identified in the search to the number of studies included in the review, ideally using a flow diagram. | Results, figure 1 |
|  | 16b | Cite studies that might appear to meet the inclusion criteria, but which were excluded, and explain why they were excluded. | NA |
| Study characteristics | 17 | Cite each included study and present its characteristics. | eTable 7-8 |
| Risk of bias in studies | 18 | Present assessments of risk of bias for each included study. | eTable 7-8 |
| Results of individual studies | 19 | For all outcomes, present, for each study: (a) summary statistics for each group (where appropriate) and (b) an effect estimate and its precision (e.g. confidence/credible interval), ideally using structured tables or plots. | Results, tables |
| Results of syntheses | 20a | For each synthesis, briefly summarise the characteristics and risk of bias among contributing studies. | Results |
|  | 20b | Present results of all statistical syntheses conducted. If meta-analysis was done, present for each the summary estimate and its precision (e.g. confidence/credible interval) and measures of statistical heterogeneity. If comparing groups, describe the direction of the effect. | Results, figure 2-3 |
|  | 20c | Present results of all investigations of possible causes of heterogeneity among study results. | Results, table 2 |
|  | 20d | Present results of all sensitivity analyses conducted to assess the robustness of the synthesized results. | Results, Table 2 |
| Reporting biases | 21 | Present assessments of risk of bias due to missing results (arising from reporting biases) for each synthesis assessed. | Results |
| Certainty of evidence | 22 | Present assessments of certainty (or confidence) in the body of evidence for each outcome assessed. | Results |
| **DISCUSSION** | | |  |
| Discussion | 23a | Provide a general interpretation of the results in the context of other evidence. | Discussion |
|  | 23b | Discuss any limitations of the evidence included in the review. | Discussion, eDiscussion |
|  | 23c | Discuss any limitations of the review processes used. | Discussion, eDiscussion |
|  | 23d | Discuss implications of the results for practice, policy, and future research. | Discussion |
| **OTHER INFORMATION** | | |  |
| Registration and protocol | 24a | Provide registration information for the review, including register name and registration number, or state that the review was not registered. | Methods |
|  | 24b | Indicate where the review protocol can be accessed, or state that a protocol was not prepared. | Methods |
|  | 24c | Describe and explain any amendments to information provided at registration or in the protocol. | NA |
| Support | 25 | Describe sources of financial or non-financial support for the review, and the role of the funders or sponsors in the review. | Discussion |
| Competing interests | 26 | Declare any competing interests of review authors. | Discussion |
| Availability of data, code and other materials | 27 | Report which of the following are publicly available and where they can be found: template data collection forms; data extracted from included studies; data used for all analyses; analytic code; any other materials used in the review. | Discussion |

**eTable4.** **PRISMA 2020 Abstract Checklist.**

| **Section and Topic** | **Item #** | **Checklist item** | **Reported (Yes/No)** |
| --- | --- | --- | --- |
| **TITLE** | | |  |
| Title | 1 | Identify the report as a systematic review. | Yes |
| **BACKGROUND** | | |  |
| Objectives | 2 | Provide an explicit statement of the main objective(s) or question(s) the review addresses. | Yes |
| **METHODS** | | |  |
| Eligibility criteria | 3 | Specify the inclusion and exclusion criteria for the review. | Yes |
| Information sources | 4 | Specify the information sources (e.g. databases, registers) used to identify studies and the date when each was last searched. | Yes |
| Risk of bias | 5 | Specify the methods used to assess risk of bias in the included studies. | Yes |
| Synthesis of results | 6 | Specify the methods used to present and synthesise results. | Yes |
| **RESULTS** | | |  |
| Included studies | 7 | Give the total number of included studies and participants and summarise relevant characteristics of studies. | Yes |
| Synthesis of results | 8 | Present results for main outcomes, preferably indicating the number of included studies and participants for each. If meta-analysis was done, report the summary estimate and confidence/credible interval. If comparing groups, indicate the direction of the effect (i.e. which group is favoured). | Yes |
| **DISCUSSION** | | |  |
| Limitations of evidence | 9 | Provide a brief summary of the limitations of the evidence included in the review (e.g. study risk of bias, inconsistency and imprecision). | Yes |
| Interpretation | 10 | Provide a general interpretation of the results and important implications. | Yes |
| **OTHER** | | |  |
| Funding | 11 | Specify the primary source of funding for the review. | No |
| Registration | 12 | Provide the register name and registration number. | Yes |

**eTable5. Moose Checklist.**

| **Criteria** | | **Brief description of how the criteria were handled in the meta-analysis** |
| --- | --- | --- |
| **Reporting of background should include** | |  |
| √ | Problem definition | No meta-analysis has assessed the proportion of negative symptoms in children and adolescents with an EOP diagnosis/at CHR-P. |
| √ | Hypothesis statement | We hypothesized that children adolescents with an EOP diagnosis/ in a CHR-P state would have frequently negative symptoms. |
| √ | Description of study outcomes | Proportion of EOP and CHR-P children and adolescents with negative symptoms. |
| √ | Type of exposure or intervention used | We included individual studies that reported the presence of negative symptoms in EOP/CHR-P in children and adolescents. |
| √ | Type of study designs used | Cross-sectional studies, longitudinal cohort and intervention studies. |
| √ | Study population | Children and adolescents with an EOP diagnosis/in a CHR-P state. |
| **Reporting of search strategy should include** | |  |
| √ | Qualifications of researchers | The credentials of the investigators are indicated in the author list and in the acknowledgements. |
| √ | Search strategy, including time period included in the synthesis and keywords | We performed a multi-step literature search using the following keywords: (“child*” OR “adolesc*” OR “paediatric” OR “pediatric” OR “teen” OR “early-onset” OR “EOP”) AND (“psychosis” OR “schizophrenia” OR “schizoaffective” OR “prodrom*” OR “ultra-high risk” OR “clinical high risk” OR “clinical high-risk” OR “attenuat*” OR “APS” OR “high risk” OR “high-risk” OR “BLIPS” OR “brief limited” OR “brief intermittent” OR “genetic high risk” OR “genetic high-risk” OR “GRD” OR “at risk mental state” OR “at-risk mental state” OR “risk of progression” OR “progression to first-episode” OR “basic symptoms”) AND “negative symptoms”. |
| √ | Databases and registries searched | PubMed and Web of Science database (Clarivate Analytics): Web of Science Core Collection. BIOSIS Citation Index. KCI-Korean Journal Database. MEDLINE. Russian Science Citation Index, and SciELO Citation Index. Cochrane Central Register of Reviews, “Schizophrenia International Research Society” (SIRS) and Early Intervention in Mental Health international conference (IEPA), clinicaltrials.gov, WHO International Clinical Trials Registry Platform (ICTRP). |
| √ | Use of hand searching | We hand-searched bibliographies of retrieved papers for additional references. |
| √ | List of citations located and those excluded, including justifications | Details of the literature search process are outlined in the results section and in the PRISMA flowchart. |
| √ | Method of addressing articles published in languages other than English | Articles in any language were selected. We received the support from international collaborators and we used translation online tools (E.g. google translator). |
| √ | Method of handling abstracts and unpublished studies | Original individual studies and conference proceedings/abstracts were included. Reviews, editorials and clinical cases were excluded. |
| √ | Description of any contact with authors | We did not contact corresponding authors to request additional data for this study. |
| **Reporting of methods should include** | |  |
| √ | Description of relevance or appropriateness of studies assembled for assessing the hypothesis to be tested | Detailed inclusion and exclusion criteria were described in the methods section. |
| √ | Rationale for the selection and coding of data | Data extracted from each of the studies were relevant to the population characteristics, study design, comparison group, exposure and outcomes. |
| √ | Assessment of confounding factors | Confounding factors were assessed as part of the meta-regressions analyses. |
| √ | Assessment of study quality. | We used a previously validated version of the Newcastle-Ottawa Scale for the evaluation of cross-sectional and cohort studies. |
| √ | Assessment of heterogeneity | Heterogeneity was assessed with the I^2^ index and sources of heterogeneity were evaluated through the sensitivity analyses. |
| √ | Description of statistical methods in sufficient detail to be replicated | Statistical methods are described in detail in the methods section. |
| √ | Provision of appropriate tables and graphics | We included the PRISMA flow-chart and several tables and graphics to describe the literature search and our results. |
| **Reporting of results should include** | |  |
| √ | Graph summarizing individual study estimates and overall estimate | We have included figures summarizing individual study estimates and overall estimates. |
| √ | Table giving descriptive information for each study included | We have presented descriptive information for each study in the supplementary material. |
| √ | Results of sensitivity testing | Subgroup analyses were conducted as specified. |
| √ | Indication of statistical uncertainty of findings | We reported 95% CI in our analyses. |
| **Reporting of discussion should include** | |  |
| √ | Quantitative assessment of bias | Publication biases were assessed with Egger test. The trim and fill method was used to correct the estimates in the case of publication biases. |
| √ | Justification for exclusion | Exclusion criteria and justification are described in the manuscript. |
| √ | Assessment of quality of included studies | Our discussion and limitations take into consideration the amount of evidence and its quality. |
| **Reporting of conclusions should include** | |  |
| √ | Consideration of alternative explanations for observed results | We discussed other explanations for our findings in the discussion section. |
| √ | Generalization of the conclusions | We have addressed the generalization of the conclusions in the discussion section. |
| √ | Guidelines for future research | We have suggested possible streams of future development and research in oyr discussion. |
| √ | Disclosure of funding source | Funding source described at the end of the manuscript. No separate funding was necessary for the undertaking of this systematic review and meta-analysis. |

**eTable6. Risk of bias (quality) assessment using modified Newcastle Ottawa Scale for cross-sectional and cohort studies.**

| **Criteria** | **Maximum Score** |
| --- | --- |
| *Cross Sectional Studies* | |
| Sample representative of target sample (e.g. all eligible or random sample)? | 2 |
| Sample size justified and satisfactory? | 1 |
| Non-response rate is defined satisfactory, and characteristics of responders/non-responders compared? | 1 |
| Ascertainment of exposure (i.e. menstrual cycle) is valid and/or well described? | 1 |
| Assessment of outcome with robust tool and/or record linkage? | 2 |
| Outcome per group reported appropriately? | 1 |
| *Cohort Studies* | |
| Representativeness of exposed cohort (e.g. total population or random sample. selected group) | 1 |
| Method used to ascertain exposure (menstrual cycle phase) is robust? | 1 |
| Exposed and unexposed are matched or adjustment for confounding factors? | 2 |
| Assessment of outcome was blind to exposure status or used record linkage. were robust tools used? | 2 |
| Follow-up period was sufficiently long for outcomes to occur (e.g. more than one menstrual cycle? | 1 |
| Loss to follow-up rate is reported. low (<30%). and same in exposed and non-exposed? | 1 |

**eTable7. Characteristics of included studies (samples with EOP individuals only).**

| **Author and year of publication** | **Country** | **Study design, (NOS score, RoB score- if applicable-)** | **Sample size EOP** | **Mean age±SD, (range), years** | **Males, %** | **Instruments used (EOP/ negative symptoms)** | **Key findings** |
| --- | --- | --- | --- | --- | --- | --- | --- |
| Alaghband-Rad 1997^5^ | USA | Cross-sectional, (4) | 29 | 10.2**±**1.5 (10-19) | N.a. | DSM-III-TR / SANS | A correlation between negative symptoms and smaller total cerebral volume was found (r=-0.65, p<0.001). Adjusting for IQ did not change the results significantly (r=0.64, p=0.002). Total cerebral volume accounted for 31% of the variance in negative symptoms (p=0.004). |
| Alfimova 2021^6^ | Rusia | Cross-sectional, (6) | 75 | 10.2**±**3.5 | 83 | ICD-10 / PANSS | The allele A of ZNF804A was associated with greater severity of negative symptoms in EOP (p<0.05). Negative symptoms were more severe in EOP than adult-onset psychosis (p< 0.05). |
| Arango 2012^7^ | Spain | Longitudinal, (6) | 61 | 15.5**±**1.7 (12-18) | 65.6 | K-SADS-Pl DSM-IV / PANSS | There was an association between left frontal CSF volume change during follow-up (r=0.58; p=0.003) and left parietal CSF change (r=0.45; p=0.03) with negative symptoms in individuals with schizophrenia. |
| Bellgrove 2006^8^ | Australia | Cross-sectional, (4) | 21 | 15.3**±**2.6 | 57.1 | K-SADS-Pl DSM-IV / PANSS | The undifferentiated schizophrenia group had higher negative symptom scores than the paranoid schizophrenia group (p=0.05). |
| Bunk 1999^9^ | Ger | Longitudinal, (3) | 44 | N.a. | 43.2 | PANSS / PANSS | Negative symptoms remained stable after 42 years (p=0.935) as opposed to positive symptoms (p<0.001) and general symptoms (p=0.002) which did improve. |
| Burton 2019^10^ | USA | Longitudinal, (5) | 318 | 17.0**±**3.3 | 62.3 | SIPS/SOPS / SIPS/SOPS | Negative symptoms were associated to poorer social functioning (p<0.001) and role functioning (p=0.003). There was an interaction between positive symptoms and negative symptoms (p=0.001) where positive symptoms decreased the association between negative symptoms and social functioning. |
| Calderon-Mediavilla 2021^11^ | Spain | Cross-sectional, (6) | 62 | 16.1**±**1.11 (13-18) | 59.7 | DSM-V/ PANSS | Negative symptoms were more severe in EOP individuals with higher levels of depression (p=0.023). A relationship was found between the dysphoria scale and some negative symptoms: social withdrawal (r=0.262; p=0.04) and active social avoidance (r=0.298;p=0.019). |
| Calvo 2014^12^ | Spain | RCT, (7, unclear risk of bias) | 55 | 16.4**±**8.0 | 61.8 | DSM-IV / PANSS | The psychoeducation group showed a greater reduction in negative symptoms after treatment compared to the non-structured group (r=0.41, p<0.05). |
| Calvo 2015^13^ | Spain | RCT, (7, unclear risk of bias) | 55 | 16.4**±**8.0 | 61.8 | DSM-IV / PANSS | After the two-year follow-up, no statistically significant differences were observed between EOP individuals in the psychoeducation group and those in the non-structured group in terms of negative symptoms (p>0.05). |
| Cheng 2021^14^ | China | Cross-sectional, (7) | 216 | 10.7**±**2.2 | 41.7 | ICD-10 / PANSS | Compared with adult-onset schizophrenia, EOP individuals had more severe negative symptoms (p=0.02). Negative symptoms at admission were predictors of poor treatment efficacy in EOP individuals (OR=0.945, p=0.009). |
| Cohen 2004^15^ | Israel | Cross-sectional, (4) | 30 | 17.1 (13-21) | 80 | DSM-IV / PANSS, BPRS | There was a significant correlation between negative symptoms and test phase response time (r=0.44, p=0.015). Individuals with higher negative symptoms responded more slowly than those with lower scores. |
| Corcoran 2005^16^ | USA | Cross-sectional, (4) | 26 | 15.0**±**1.8 | 61.5 | DSM-III-R / PANSS | IQ and negative symptoms highly associated with one another (r=0.63, p=0.003). Smell identification deficits were significantly associated with greater negative symptoms (r=0.47, p=0.03), including blunted affect (r=0.51, p=0.02). |
| Correll 2005^17^ | USA | Longitudinal, (5) | 29 | 16.2**±**2.7 (12-22) | 65 | DSM-IV / SIPS/SOPS/ K-SADS-E | 38.4% of individuals had only attenuated negative symptoms or no psychotic symptoms at 6 months follow-up. Brief psychotic disorder was associated with lower maximum levels of negative symptoms compared to psychosis NOS (p=0.02). |
| Bahn 2002^31^ | Korea | Cross-sectional, (4) | 48 | 17.9**±**2.7 | 60.4 | DSM-III-R / PANSS | EOP individuals diagnosed with schizophrenia showed negative symptoms more frequently than late onset schizophrenia (p<0.05). 77% EOP individuals had negative symptoms. |
| Correll 2008^18^ | USA | Longitudinal, (5) | 26 | 15.9**±**2.6 | 65.4 | DSM-IV / SOPS | A correlation was found between higher negative symptoms and shorter follow-up duration (p=0.012). |
| Correll 2022^19^ | USA | RCT, (6, low risk of bias) | 326 | 15.3**±**1.4 | 63.8 | DSM-IV / PANSS | After six weeks, the treatment-naive group did not improve in negative symptoms with lurasidone compared to placebo (p=0.18; ES=0.40). In the previously treated group, treatment with lurasidone was associated with greater improvement versus placebo in negative symptoms (p=0.017; ES=0.32). |
| Craddock 2018^20^ | USA | Cross-sectional, (5) | 124 | 13.3**±**2.7 | 52 | DSM-III-R or DSM-IV / PANSS | Using a two-factor solution containing positive and negative dimensions 29.8% EOP individuals had low scores on both dimensions, 26.6% high negative scores with low positive scores and 44.0% high scores on both dimensions. |
| DeVylder 2013^21^ | USA | Cross-sectional, (7) | 84 | 14.7**±**2.3 | 57.1 | DSM-IV / N.a. | Negative symptoms were increased in the EOP individuals with declining social support compared to the stable social support group (p<0.05). |
| Downs 2019^22^ | UK | Longitudinal, (6) | 638 | (10.0-17) | 51.6 | ICD-10 / PANSS, NLP | The presence of negative symptoms at first episode was significantly associated with multiple treatment failure (adjusted HR=1.62, p=0.02). |
| Duan 2021^23^ | China | Cross-sectional, (5) | 36 | 15.5**±**1.8 (12-18) | 44.4 | DSM-IV-TR / PANSS | Significant negative correlations between the bilateral hippocampal volumes and total negative symptoms (p<0.025). Impairments in verbal memory had an indirect effect mediating the association between negative symptoms in the left hippocampal volume (bootstrap estimate effect=−0.0040) and right hippocampal volume (bootstrap estimate effect=−0.0034). |
| Fields 1994^24^ | USA | Cross-sectional, (4) | 34 | (6.0-16.0) | 61.8 | KIDDIE-PANSS / PANSS | Negative symptoms were more frequent in EOP individuals with schizophrenia admitted into the hospital than in other psychosis (p=0.04). |
| Findling 2003^25^ | USA | Other intervention study (3) | 16 | 13.8**±**1.5 (12-17) | 75 | DSM-IV / PANSS | Negative symptoms improved after treatment with olanzapine in 8-week, open-label, outpatient study (p<0.01). This difference was significant since week six (p<0.01). |
| Fleischhaker 2005^26^ | Germany | Longitudinal, (4) | 81 | n.a. | 55.5 | DSM-III-TR / SANS | EOP individuals with a family history of schizophrenia or schizoaffective disorder compared to those without a family history of schizophrenia or schizoaffective disorder did not have higher scores of negative symptoms (p=0.32). |
| Fraguas 2014^27^ | Spain | Longitudinal, (5) | 66 | 16.2**±**1.64 | 71.2 | DSM-IV / PANSS | Negative symptoms at baseline were associated to executive performance at baseline (β=−4.811, p=0.007). Negative symptoms at baseline predicted an improvement in executive performance after two years (β=4.688, p=0.008). |
| Garcia Traverso 2019^28^ | Spain | Longitudinal, (4) | 110 | 15.5 | N.a. | N.a. / PANSS | Negative symptom dimension was the most consistent and stable component. It was predominant at all but one assessment, accounting for 23.66% of overall variance at baseline, 25.7% at 4 weeks, 13.9% at 6 months 36.3% at 12 months and 40.1% at 24 months. |
| Garcia-Amador 2010^29^ | Spain | Cross-sectional, (3) | 49 | 15 | N.a. | N.a. / PANSS | A higher improvement in negative symptoms correlated with a thinner frontal cortex at baseline (r=0.5, p=0.003). Reduction in negative symptoms correlated with baseline negative symptom severity (r=0.6, p<0.001). |
| Garcia-Amador 2020^30^ | Spain | Longitudinal, (3) | 49 | 15 | N.a. | N.a. / PANSS | Reduction in negative symptoms was positively correlated with baseline negative symptom severity (r=0.6, p< 0.001). Reduction in negative symptoms was positively correlated to frontal cortical thickness (r=0.5, p=0.003). This correlation remained significant after controlling for baseline negative symptom severity (r=0.4, p=0.003). |
| Gerstenberg 2015^32^ | Switzerland | Cross-sectional, (4) | 12 | 14.9**±**1.2 (9.0-19.0) | 58.3 | DSM-IV / PANSS | No association found between negative symptoms and sleep spindle density (p=0.07). |
| Gothelf 2003^33^ | Israel | Other intervention study, (5) | 43 | 14.9**±**1.7 | 62.8 | DSM-IV / PANSS | A significant improvement in negative symptoms was observed after eight weeks: average decline in negative symptoms scores from baseline to week 8 was 14.0% for risperidone, 17.7% olanzapine and 19.2% haloperidol. |
| Hadjez 2003^34^ | Israel | Cross-sectional, (3) | 20 | 16.2**±**2.8 | N.a. | K-SADS-DSM-IV / PANSS | A correlation was found between negative symptoms and emotional expression (r=0.58, p<0.01), involvement (r=0.54, p<0.05) and recall (r=0.48, p<0.05). |
| Hassan 2011^35^ | Egypt | Longitudinal, (5) | 56 | 17**±**3.7 | 46.4 | DSM-IV / PANSS | More impairment in premorbid functioning (p<0.01) and global functioning (p<0.01) were associated with more negative symptoms (p<0.01). The remittent group had less severe negative symptoms (p=0.024). |
| Hemmerle 2010^36^ | German | Other intervention study, (3) | 24 | 16.9**±**1.2 (14-19) | 66.7 | ICD-10 / PANSS | Participants attending a programme of residential outpatient care following discharge from a clinic showed a significantly greater decrease on negative symptoms than the control group (p=0.002). |
| Hintze 2015^37^ | Poland | Cross-sectional, (5) | 33 | 17.3**±**1.2 (15-19) | 60 | ICD-10 / PANSS | No significant differences between EOP individuals with and without a family burden were observed in negative symptoms. A greater intensity of negative symptoms correlated with a lower performance on the WCST evaluating executive functions and working memory (higher percentage of perseverative errors) (r=0.38, p<0.05). Greater intensity of negative symptoms correlated with less % numbers correct and more % of wrong numbers as per the N-back test (r=-47 and r=0.47 both p<0.001). |
| Hollis 2003^38^ | UK | Cross-sectional, (7) | 110 | 14.7**±**1.5 (10-17) | 53 | DSM-III-TR / OPCRIT | The negative symptom dimension was associated with enuresis (OR=1.93, p<0.05) and incontinence during psychosis (OR=3.35, p=0.005). |
| Jarbin 2003^39^ | Sweden | Longitudinal, (5) | 68 | 15.7 (11.8-18.7) | 48.5 | DSM-IV / PANSS | Individuals with stable schizophrenia spectrum disorder had more severe negative symptoms than those with stable affective psychotic disorders (p<0.01). Those whose diagnosis changed to schizophrenia spectrum disorders after a follow up of 10.5 years average had more severe negative symptoms than those with stable affective psychotic disorders (p<0.01) but less severe negative symptoms than those with stable schizophrenia spectrum disorders (p<0.05). |
| Jarbin 2004^40^ | Sweden | Longitudinal, (6) | 88 | 15.7**±**1.5 | 50 | DSM-IV / PANSS | EOP individuals who never attempted suicide had more negative symptoms during first episode (p<0.05). |
| Kafali 2019^41^ | Turkey | Cross-sectional, (5) | 30 | 16.3**±**1.8 (13-19) | 40 | K-SADS-PL DSM-IV / PANSS | The presence of ≥1 negative symptom (OR=1.3, 95% CI=0.5–93.6) and ≥2 negative symptoms (OR=2.75, 95% CI=0.8–9.4) were higher in the EOP than in the BD group. 53.5% EOP had negative symptoms. |
| Karacetin 2022^42^ | Turkey | Cross-sectional, (8) | 101 | 16.3**±**1.3 | 65.3 | DSM-IV-TR / PANSS | Valine/Valine group had a higher score of negative symptoms compared to Valine/Metionine group (p=0.018, d=0.55). |
| Karakus 2022^43^ | Turkey | Longitudinal, (7) | 132 | 14.9**±**1.7 | 33.3 | DSM-V / PANSS | Age at onset was lower in those with persistent negative symptoms (p=0.022). The % of history of suicide attempts was higher in those without persistent negative symptoms (p=0.002). Duration of untreated psychosis was higher in those with persistent negative symptoms (p=0.022). |
| Kemp 2013^44^ | USA | RCT, (6, high risk of bias) | 107 | 12.8**±**3.08 | 62.6 | DSM-IV / PANSS | Less severe negative symptoms were associated with obesity status at baseline (p=0.003). |
| Kim 2009^45^ | Republic of Korea | Other intervention study, (2) | 22 | 14.0**±**2.4 (6.5-16.8) | 54.5 | DSM-IV / N.a. | A greater general response to aripiprazole according to the Clinical Global Impression–Improvement scale was found in those individuals with negative symptoms than in those with positive symptoms (p=0.028; r=–0.47). |
| Kim 2013^46^ | Korea | Cross-sectional, (3) | 16 | 16.2**±**1.8 | 50 | DSM-IV-TR / PANSS | Early onset group also exhibited more negative symptoms (p=0.022) and of more severity (p=0.007) than adult-onset group. There was a correlation between an earlier age of onset with number of negative symptoms (p=0.022) and severity of negative symptoms (p=0.007). |
| Kumra 1996^47^ | USA | RCT, (3, high risk of bias) | 21 | 14.0, 2.4 (6-18) | 52.4 | DSM-IIIR / SANS | Clozapine decreased negative symptoms compared to haloperidol in double blind RCT (p=0.002). |
| Kumra 1998^48^ | USA | RCT, (3, high risk of bias) | 23 | 16.0**±**4.0 | 52.2 | DSM-IIIR / SANS | At week 8 of olanzapine, relative to baseline, there was a 21% improvement in negative symptoms (no comparison group, open label). |
| Kumra 2008^49^ | USA | RCT, (3, high risk of bias) | 39 | 15.6**±**2.1 (10-18) | 53.8 | DSM-IV / SANS | Clozapine was more efficacious in decreasing negative symptoms than olanzapine after 12 weeks (p=0.02, d=0.92). |
| Li 2020^50^ | China | Cross-sectional, (6) | 72 | 14.7**±**1.8 | 33.3 | SCID-DSM-IV / PANSS | A negative correlation was found between negative symptoms and abnormal Dynamic amplitude of low-frequency fluctuations (dALFF) variability of the right middle temporal gyrus (r=-0.2723, p=0.0207). |
| Maki 2008^51^ | Finland | Longitudinal, (1) | n.a. | n.a. | N.a. | N.a. / N.a. | 94% of subjects who got psychosis reported negative symptoms. Negative symptoms were associated with the onset of psychosis. |
| Matsumoto 2001^52^ | UK | Cross-sectional, (4) | 40 | 15.5**±**2.2 (8-17) | 50 | DSM-IV / PANSS | No correlation between whole brain volume and EOP individuals’ negative (r=0.2, p=0.1) symptoms was found. A correlation was found between negative symptoms score and left (r=-0.2. p=0.03) and right (r=-0.02, p=0.04) hippocampal volumes in MRI. |
| McClellan 1999^53^ | USA | Longitudinal, (6) | 51 | 14.9**±**2.0 (9-17) | 65 | DSM-IV / SANS | Individuals with schizophrenia (72%) and schizoaffective disorder (43%) had more negative symptoms at baseline than those with bipolar disorder (21%) or psychosis-NOS (9%). |
| McClellan 1999^54^ | USA | Longitudinal, (6) | 54 | 14.8**±**2.1 | 64.8 | DSM-IV / SANS | Negative symptoms upon entry predicted lower highest level of functioning at one year (β=0.6, p=0.005) and two year follow up (β=0.48, p=0.003). |
| McClellan 2002^55^ | USA | Longitudinal, (6) | 69 | 14.8**±**2.2 | 60.86 | DSM-IV / SANS | IQ was inversely related to negative symptoms (r=–0.41, p<0.05). Those taking antipsychotic agents had more severe negative symp-toms (p<0.01), while those taking lithium had less severe negative symptoms (p<0.005). Negative symptoms upon entry predicted highest level of functioning at one year (β=0.62, p<0.001) and two year follow up (β=0.55, p<0.001)- |
| McClellan 2003^56^ | USA | Longitudinal, (4) | 69 | 14.9**±**2.2 (7-18) | 68 | DSM-IV / PANSS | A correlation was found between introversion on the sociability scale and negative symptoms (r=0.45, p<0.005). A correlation was found between poorer peer relationships and negative symptoms (r=0.26, p<0.005). |
| McConville 2000^57^ | USA | Other intervention study, (1) | 10 | 13.1 (12-17) | 10 | DSM-IV (SCID) / SANS | Negative symptoms improved after 20 days in open-label trial with quetiapine (p=0.0006). |
| McConville 2003^58^ | USA | Other intervention study, (2) | 10 | 13.1 (12-17) | 10 | DSM-IV (SCID) / SANS | Negative symptoms improved in 88-week, open-label trial with quetiapine (p<0.05). |
| Meng 2006^59^ | Multi-country | Cross-sectional, (5) | 56 | 16.2**±**1.4 (12-18) | 51.1 | DSM-IV (SCID) / PANSS | Social functioning was associated with negative symptoms (r=-0.42, p=0.006). More negative symptoms at follow-up were associated with more negative symptoms at baseline (p<0.05) but not with age or gender. |
| Merchan-Naranjo 2007^60^ | Spain | Longitudinal, (2) | 24 | <18 | N.a. | DSM-IV/ PANSS | Negative symptoms at baseline were the only significant variable that predict the functional outcome at the two-year follow-up (p=0.010). |
| Morch-Johnsen 2022^61^ | Norway | Cross-sectional, (7) | 169 | 15.5**±**1.5 (12-18) | 59.2 | DSM-IV (SCID) / PANSS | Negative symptom scores for both apathy (β=−0.257, p=0.002) and diminished expression (β=−0.259, p=0.001) were inversely associated with verbal learning scores. An association was also seen between diminished expression and speed of processing (β=−0.173, p=0.024). |
| Mozes 2006^62^ | Israel | Other intervention study, (4) | 25 | 11.1**±**1.6 (9-14) | 40 | DSM-IV / PANSS | The percentage of children achieving greater than 50% reduction in negative PANSS scores was higher in olanzapine (41.7%) than in risperidone (7.7%) (p=0.047); however, this difference did not remain significant following Bonferroni correction. No difference in symptoms when evaluated continuously (p=0.144). |
| Mueller 2020^63^ | Germany | Other intervention study, (6) | 25 | 17.5**±**1.5 (14-20) | 56 | DSM-IV / PANSS | No differences between CBT+ TAU and TAU in negative symptoms at baseline (p=0.317), post treatment at 9 months follow-up (p=0.169) or at 18 months follow-up (p=0.086). |
| Nechmad 2003^64^ | Israel | Cross-sectional, (5) | 50 | 17.0**±**2.1 | 64 | DSM-IV / SANS | Individuals with schizophrenia and OCD scored higher on the SANS subscale for affective flattening or blunting than those without OCD (p=0.003). No overall differences in negative symptoms (p=0.35). |
| Oades 2006^65^ | Germany | Longitudinal, (5) | 28 | 17.5**±**0.4 | 75 | DSM-IV / SANS | The amplitude event-related brain response mismatch negativity was associated with negative symptoms. |
| Paillere-Martinot 2000^66^ | France | Longitudinal, (5) | 36 | 17.5**±**1.8 (14-21) | 50 | DSM-III-R / SANS | Negative symptoms were higher in individuals with depression (p=0.006) at baseline than in non-affective psychosis. Individuals who developed schizophrenia had more residual negative symptoms at discharge than those with affective psychosis (p=0.03). |
| Pandina 2000^67^ | Multi-country | Other intervention study, (4) | 324 | 15.4**±**1.4 (12-17) | 62.7 | DSM-IV / PANSS | Correlations between speed of processing and negative symptoms at baseline (r=0.309, p<0.05) and 6 months follow up (r=0.184, p<0.05). |
| Parellada 2006^68^ | Spain | Other intervention study, (3) | 50 | 16 (<18) | N.a. | DSM-IV / PANSS | No differences were found between quetiapine and olanzapine in negative symptoms (p>0.05). There was a significant decrease in negative symptoms in both groups (p<0.05). |
| Parellada 2015^69^ | Spain | Longitudinal, (7) | 83 | 15.5**±**1.8 (9-17) | 69 | DSM-IV (KSADS) / PANSS | Primary negative symptoms at 2 years are more prominent in EOP individuals with an initial diagnosis of schizophrenia (compared with BD p=0.002 and other psychosis p=0.003). Primary negative symptoms at 2 years are more prominent in EOP individuals with lower baseline general symptoms (r=-0.242, p=0.043) and more prominent negative symptoms at baseline (p=0.025). |
| Paruk 2017^70^ | South Africa | Cross-sectional, (5) | 45 | 15.9**±**1.8 (10-18) | 68.9 | DSM-IV-TR / PANSS | No significant association between negative symptoms and a family history of psychosis was found (p>0.05). |
| Pencer 2005^71^ | Canada | Longitudinal, (5) | 69 | 17.3**±**1.2 (15-19) | 71 | DSM-IV / PANSS | After two years, 51.0% EOP were in remission from negative symptoms. Negative symptoms were significant predictors of quality of life (p<0.001). |
| Perez-Garza 2016^72^ | Mexico | Longitudinal, (4) | 87 | 14.9**±**1.5 (12-17) | 69 | DSM-IV / PANSS | There was a significant improvement in negative symptoms at 3 and 6 months follow up (𝑝<0.001) but no gender effect (p>0.05). |
| Petruzzelli 2018^73^ | Italy | Cross-sectional, (5) | 60 | <18 | 61.7 | DSM-IV-TR / PANSS, SANS | The four factors model we presented highlights “negative symptoms” as the most consistent factor (blunted affect (N1), emotional withdrawal (N2), lack of spontaneity and flow conversation (N6), active social avoidance (G16), poor rapport (N3), passive/apathetic social withdrawal (N4). |
| Poyurovsky 2008^74^ | Israel | Cross-sectional, (6) | 44 | 16.6**±**1.6 (13-18) | 56.8 | SCID DSM-IV / SANS | No association between OCD symptoms and negative symptoms were found (all p>0.05) |
| Puig 2012^75^ | Spain | Cross-sectional, (5) | 45 | 16.9**±**1.5 (12-18) | 48.9 | DSM-IV-TR / PANSS | Correlations between negative symptoms and daily living skills/ functioning were found (r=-0.348 p<0.05). |
| Ramanathan 2015^76^ | USA | Cross-sectional, (6) | 58 | 13.7**±**2.4 (10-18) | 50 | SCID-DSM-IV / SIPS/SOPS | There was a statistically significant association between negative symptoms in males and a delayed age of puberty (p=0.001) as compared to early maturers. |
| Rapado-Castro 2010^77^ | Spain | Longitudinal, (5) | 99 | 15.5**±**1.8 (7-17) | 66.7 | DSM-IV / PANSS | Negative dimension was the most consistent and stable over time and was predominant at baseline (23.9%) and at 4 weeks (25.7%). |
| Rapado-Castro 2019^78^ | Spain | RCT, (6, high risk of bias) | 22 | 16.3**±**1.2 (14-18) | 59 | DSM-IV-TR / PANSS | An association was found between improvements in executive functioning performance and a reduction in negative symptoms (r=0.758, p=0.029 for semantic; r=–0,733, p=0.025 for verbal fluency). Negative correlations were found between increased verbal fluency (r=-0.73, p=0.025) and a decrease in negative symptoms in the psychoeducation group. |
| Reddy 1996^79^ | South India | Cross-sectional, (2) | 43 | 15**±**1.4 (10-16) | 39.5 | ICD-9/10 / ICD-10 DCR | Negative symptoms appeared in 46.5% EOP individuals with schizophrenia. |
| Reig 2011^80^ | Spain | Longitudinal, (7) | 92 | 15.7**±**1.7 (9-18) | 69.6 | DSM-IV (KSADS-PL) / PANSS | In male individuals, the correlation analysis revealed a significant negative relationship between negative symptom and whole-brain GM volume (r=0.292, p=0.025). In the region of interest analyses of GM volumes, this correlation was significant only in the parietal lobe (right: r=0.399, p=0.002; left: r=0.296, p=0.023). |
| Remberk 2012^81^ | Poland | Cross-sectional, (4) | 32 | 16.7**±**1.5 (13-18) | 46.9 | ICD-10 / PANSS | Negative symptoms correlated with perseverative errors (r=0.31, p<0.05), phonological fluency (r=−0.27, p<0.05) and number of uncommon responses (r=0.27, p<0.05). |
| Remberk 2012^82^ | Poland | Cross-sectional, (6) | 104 | 16.7**±**1.2 (13.4-19.1) | 47.1 | ICD-10 / PANSS | A correlation was found between negative symptoms and length of hospitalization (r=0,194, p=0.004). In schizophrenia more negative symptoms then in acute and transient psychotic disorders were detected (p=0.006). |
| Remberk 2015^83^ | Poland | Longitudinal, (4) | 63 | 16.6**±**1.2 (14.1-19.1) | 50.8 | ICD-10 / PANSS | Negative symptoms were related to mental health service utilization during the follow-up. Severity of negative symptoms was associated with diagnosis of schizophrenia at baseline (β=0.287, p=0.007). A higher level of negative symptoms was associated with a higher probability of receiving pharmacotherapy (β=0.243, p=0.025). The drug-free subgroup with no occupational/educational activity compared with the drug-treated subjects showed lower levels of baseline negative symptomatology (p<0.05). |
| Ross 2013^84^ | USA | Other intervention study, (3) | 19 | 10.5**±**2.4 (6-15) | 73.7 | DSM-IV / SANSS | Negative symptoms improved in EOP individuals on olanzapine in this open label trial after a year (p=0.004). |
| Salazar de Pablo 2021^85^ | Spain | Longitudinal, (5) | 108 | 15.5**±**1.8 (7-17) | 68.5 | DSM-IV / PANSS | Negative symptoms were more severe in EOP with non-affective psychosis than affective psychosis (p=0.045). |
| Savitz 2015^86^ | Multi-country | RCT, (7, low risk of bias) | 174 | 15.3**±**1.5 (12-17) | 66 | DSM-IV / PANSS | No difference found in efficacy for negative symptoms between paliperidone and aripiprazole after two months (p=0.535) or six months (p=0.696). |
| Schwartz-Stav 2006^87^ | Israel | Cross-sectional, (4) | 32 | 17.5 (13-19) | 62.5 | K-SADS-DSMIV / PANSS | A correlation was found between negative symptoms and general unawareness (r=0.48) but not to unawareness for psychotic symptoms (p>0.05). |
| Shaw 2006^88^ | USA | RCT, (5, unclear risk of bias) | 25 | 12.3**±**2.4 (7-16) | 60 | DSM-IV / SANS | Clozapine was more efficacious than olanzapine for negative symptoms (p=0.04; ES=0.89) after eight weeks. |
| Şimşek 2016^89^ | Turkey | Cross-sectional, (4) | 30 | 14.7**±**1.9 (10-17) | 39 | K-SADS-PL / PANSS | There was a significant correlation between decreased IL-10 and increased IL-4 levels with increased negative symptoms (r=-0.65, p=0.02 and r=0.67, p=0.02, respectively). |
| Slosarczyk 2022^90^ | Poland | Longitudinal, (5) | 69 | 16 (14-19) | 49.3 | DSM-IV-TR / N.a. | Premorbid sadness was associated to higher severity of negative symptoms at baseline (p<0.05). Poor emotional expression primarily correlated with later apathy/abulia (p<0.05). |
| Tang 2012^91^ | China | Longitudinal, (5) | 29 | 16.1**±**0.9 (12-19) | 44.8 | DSM-IV-TR / PANSS | The loss of grey matter volume did not correlate with negative symptoms (p>0.05). |
| Thaden 2006^92^ | USA | Cross-sectional, (6) | 59 | 16**±**2.1 (7-18) | 59.3 | DSM-IV / SANS | No significant correlation was found between attentional capacity and negative symptoms (p>0.05). |
| Thakur 2003^93^ | India | Cross-sectional, (7) | 101 | 16.1**±**1.7 (6-18) | 66.3 | DSM-IV / SANS | From four factors generated, two of them focused on negative symptoms: primary negative factor including unchanging facial expression, decreased spontaneous movements, paucity of expressive gestures, poor eye contact, poverty of speech, grooming and hygiene and impersistence) and secondary negative factor (affective non-responsivity, inappropriate affect, lack of vocal inflections, physical anergia, reduced recreational interest and activities, decreased ability to feel intimacy and closeness, poor interpersonal relationships). Individuals in the primary negative group having poor educational background (p=0.002) and individuals in the secondary negative group showed longer illness duration (p<0.001). |
| Vines 2022^94^ | USA | Longitudinal, (5) | 13 | 17.8**±**2.8 (12-25) | 48.9 | SCID-DSM-IV, SIPS/SOPS / SIPS/SOPS | Greater negative symptom severity was associated with less cognitive reappraisal (β=0.27, p=0.02) and greater overall emotion regulation impairment (β=0. 31, p=0.02). |
| Vyas 2007^95^ | UK | Longitudinal, (3) | 40 | 15.6**±**2.3 | 50 | DSM-IV / PANSS | Psychosocial outcome as per the Social Adaptation Self-Evaluation Scale correlated negatively with negative symptoms (r=0.45, p=0.04). Functioning scores did not correlate with negative symptoms (p=0.17). |
| Wang 2017^96^ | China | Longitudinal, (5) | 35 | 15.5**±**1.8 (12-18) | 57.1 | DSM-IV-TR / PANSS | The precuneus with reduced global functional connectivity density is significantly negatively correlated with negative symptoms in the slow-4 band (p<0.05) |
| Wang 2020^97^ | China | Cross-sectional, (6) | 39 | 15.5**±**1.8 (12-18) | 57.1 | DSM-IV-TR / PANSS | The intrinsic functional connectivity and structural properties of the left frontal white matter correlated with negative symptoms in EOP (p<0.05). Negative symptoms were negatively correlated with reduced fiber counts through the frontal white matter (r=-0.39, p=0.043). |
| Wedervang-Resell 2020^98^ | Norway | Cross-sectional, (4) | 39 | 16.3**±**1.4 (12-18) | 66.7 | KSADS-PL / PANSS | Compared with the mild negative group (PANSS Neg <21), the Severe Negative group (p ≥21) had significantly longer DUP (p=0.001) and decreased C-GAS scores (p=0.03). The levels of triglycerides were significantly higher in the severe negative than in the mild negative individual group (p=0.032). |
| Wen 2011^99^ | China | Cross-sectional, (5) | 29 | 14.9**±**1.6 (<18) | 34.5 | DSM-5 / PANSS | Negative symptoms were less severe in EOP than in CHR-P individuals fulfilling DSM-5-APS criteria (p=0.0002). Gray matter volume of the hippocampus was negatively correlated with negative symptoms in EOP (r=-0.474, p=0.001) |
| Wozniak 2008^100^ | USA | Longitudinal, (3) | 36 | 14.6**±**1.9 (10-17) | 64 | DSM-IV / PANSS | Baseline IQ was correlated with negative (r=-0.706, p=0.007), symptoms at follow-up. No correlation between IQ and negative symptoms (r=-0.212, p=0.228) at baseline. |
| Yang 2014^101^ | China | Cross-sectional, (5) | 26 | 14.5**±**1.9 (7.5-17.9) | 50 | DSM-IV / PANSS | EOP individuals with more predominant negative symptoms are associated with abnormality in the superior temporal gyri-inferior frontal gyri network (p<0.05.) |
| Zakowicz 2022^102^ | Poland | Longitudinal, (5) | 31 | 14**±**1.9 (11-18) | 48.4 | DSM-IV / PANSS | Evaluating the correlation between language Embodiment and negative symptoms, a strong correlation was observed between negative symptoms and the "Thought, Language, and Communication Scale" (TLCS (r=0.63, p<0.01) but not Zabór Verbal Task (ZBT, p<0.05). |
| Zheng 2016^103^ | China | Longitudinal, (4) | 35 | 15.5**±**1.6 (12-18) | 57.1 | DSM-IV-TR / PANSS | Voxel-wise amplitude of low-frequency (0.01–0.08 Hz) fluctuations in the precuneus had a significant negative correlation with PANSS negative symptom scores (r=-0.44, p=0.0067). |
| Zhou 2022^104^ | China | Cross-sectional, (5) | 20 | (13-18) | 35 | DSM-IV / PANSS | Whole-brain correlation analysis found that the degree centrality in the right superior parietal lobe (SPL) was positively correlated with PANSS-negative symptom scores (r=0.79); degree centrality in the left precuneus was positively correlated with self-certainty) scores (r=0.70); and degree centrality in the left medial frontal gyrus was negatively correlated with self-reflectiveness (scores (r=0.69). |
| APS: Attenuated Psychosis Syndrome; BD: Bipolar Disorder; BPRS: Brief Psychiatric Rating Scale; CBT: Cognitive behavioural therapy; C-GAS: Children's Global Assessment Scale; CSF: Cerebrospinal Fluid; DSM: Diagnostic and Statistical Manual of Mental Disorders; EOP: early onset psychosis; ES: effect size; GAF: global assessment of functioning; GM: gray matter; HR: hazard ration; ICD: International Classification of Diseases; IL: interleukin; IQ: Intelligence quotient; K-SADS-PL: Kiddie Schedule for Affective Disorders and Schizophrenia – Present and lifetime; OR: odds ratio; N.a. no available; NLP: nature language processing; NOS: not otherwise specified; OCD: obsessive compulsive disorder; OPCRIT: Operational Criteria Checklist for Psychotic Illness and Affective Illness; PANSS: Positive and Negative Syndrome Scale; RCT: Randomised Clinical Trial; SANS: Scale for the Assessment of Negative Symptoms; SCID: Structured Clinical Interview for DSM Disorders; SIPS: Structured Interview of Psychosis-risk Syndromes; SOPS: Scale of Prodromal Symptoms; TAU: treatment as usual; WCST: Wisconsin Card Sorting Test. | | | | | | | |

**eTable8**. **Characteristics of included studies (samples with CHR-P individuals)**.

| **First author** | **Country** | **Study design (NOS score, RoB score- if applicable-)** | **Sample size CHR-P** | **Mean age±SD, (range), years** | **Males, %** | **Instruments used (EOP/ Negative symptoms)** | **Key findings** |
| --- | --- | --- | --- | --- | --- | --- | --- |
| Addington 2014^105^ | Multi-country | Cross-sectional, (6) | 155 | 17.8**±**3.7 (13-35) | 52.3 | SIPS/SOPS / SIPS/SOPS | A correlation between depressive symptoms and negative symptoms was found (r=0.38, p<0.01). |
| Amminger 2010^106^ | Austria | RCT, (7, low risk of bias) | 81 | 16.4**±**2 (13-25) | 33.5 | DSM-IV / PANSS | The omega-3 group had significantly lower negative symptoms scores at 12 weeks (p<0.05), 6 months (p<0.05), and 12 months (r=0.52, p<0.05) than the control cohort. |
| Amminger 2015^107^ | Austria | RCT, (5, low risk of bias) | 80 | 16.4**±**2.1 (13-25) | 32.5 | PANSS / PANSS | More severe baseline negative symptoms were associated with treatment response in the ω-3 supplemented group compared to the control group (Cohen’s d=0.7) |
| Armando 2012^108^ | Italy | Cross-sectional, (7) | 111 | 16.6**±**1.5 | 36.9 | PANSS / PANSS | There was a significant group difference in the PANSS negative subscale (p=0.0081), with higher scores in CHR-P individuals with 22q11 compared to those without it. After controlling for the influence of IQ and depressive symptoms, differences remained (p=0.015, p=0.034). |
| Bartholomeusz 2014^109^ | Austria | Cross-sectional, (4) | 39 | 16.4**±**2.6 (13-25) | 35.89 | PANSS / PANSS | A correlation was found between increased left amygdala volume and negative symptoms in females (p=0.020) but not males. |
| Boldrini 2020^110^ | Italy | Cross-sectional, (4) | 26 | 15.5**±**1.4 | 46.2 | SIPS/SOPS / SIPS/SOPS | A positive correlation was found between psychotic-level defences and negative symptoms including social anhedonia (r=0.630; p<0.01), avolition (r=0.504; p<0.01), expression of emotions (r=0.510; p<0.01), and experience of emotion and self (r=0.500; p<0.01). |
| Gerstenberg 2020^111^ | Germany | Cross-sectional, (4) | 21 | 15.0**±**1.4 (12-17) | 47.6 | DSM-IV / SIPS/SOPS | Poorer current global functioning (r=−0.26; p=0.015)., social functioning (r=−0.38, p=0.0007) and role functioning (r=−0.25; p=0.025) were associated with more severe negative symptoms. |
| Giordano 2022^112^ | Italy | Longitudinal, (6) | 71 | 13.9**±**2.1 (9-17.6) | 59.1 | SIPS/SOPS / SIPS/SOPS | 100% CHR-P experienced negative symptoms of at least moderate severity. Negative symptoms at baseline did not predict transition to psychosis (p=0.76). |
| Lencz 2004^113^ | USA | Cross-sectional, (5) | 42 | 16.3**±**2.3 | 63.4 | SIPS/SOPS / SIPS/SOPS | No differences in negative symptoms found between at risk individuals with attenuated negative/disorganized symptoms only, CHR-P subjects with attenuated positive symptoms and subjects with schizophrenia-like psychosis (p>0.05). |
| Lindgren 2010^114^ | Finland | Cross-sectional, (5) | 62 | 16.6**±**0.9 (14-19) | 21 | SIPS/SOPS / SIPS/SOPS | Negative symptoms correlated negatively with processing speed (r=−0.31, p=.014) and verbal performance (r=−0.37, p=0.03). |
| Lindgren 2021^115^ | Finland | Longitudinal, (6) | 50 | 16.5 (15-18) | 21.6 | SIPS/SOPS / SIPS/SOPS | Negative symptoms predicted transition to psychosis (AUC=0.74, p<0.01). |
| McGorry 2013^116^ | Austria | RCT, (7, unclear risk of bias) | 115 | 17.9**±** (14-30) | 39.4 | CAARMS / SANS | No significant differences in negative symptoms between the CBT+ risperidone, CBT+ placebo, supportive therapy+placebo and monitoring groups (p>0.05). |
| Miklowitz 2014^117^ | Multi-country | RCT, (6, high risk of bias) | 129 | 17.4**±**4.1 (12-35) | 57.4 | SIPS/SOPS / SIPS/SOPS | Family-Focused Treatment was not associated to an improvement in negative symptoms (p>0.05). CHR-P individuals who entered the trial on antipsychotics showed greater improvement in total negative symptom scores than those not on antipsychotics (p=0.03). The improvements in total negative symptom scores were not attributable to changes in specific negative symptom items (p=0.90). |
| Niendam 2006^118^ | USA | Longitudinal, (5) | 45 | 17.7**±**4.0 (12-19) | 64 | SIPS/SOPS / SIPS/SOPS | More severe negative symptoms were associated with poorer social functioning (r=0.47, p=0.001). No correlation between negative symptom score and neurocognitive measures was found (p>0.05). |
| O’Brien 2006^119^ | USA | Longitudinal, (3) | 26 | 16.2 (12-35) | 53.8 | SIPS/SOPS / SIPS/SOPS | The largest number of critical comments (39%) from the family referred to negative symptoms, particularly social withdrawal (7%) and lack of motivation (32%). 89% of the sample either improved or remained the same on the negative symptom scale. |
| O’Brien 2008^120^ | USA | Cross-sectional, (3) | 32 | 15.7 (12-35) | 60 | DSM-IV / SIPS/SOPS | Positive remarks offered by family members predict a decrease in negative symptoms (p<0.05). |
| Pelizza 2018^121^ | Italy | Longitudinal, (5) | 36 | 15**±**1.4 (13-18) | 50 | CAARMS / PANSS, CAARMS | No significant differences found between CHR-P and EOP individuals in negative symptoms including observed blunted affect, alogia and anhedonia (all p>0.05). |
| Pelizza 2020a^122^ | Italy | Longitudinal, (6) | 90 | 15.9**±**1.6 (13-18) | 54.4 | CAARMS/ PANSS, CAARMS | Difficulties in metacognition- cognition related to cognitive impairments- had significant positive correlations with negative symptoms dimensions (p=0.0001). |
| Pelizza 2020b^123^ | Italy | Longitudinal, (5) | 76 | 15.8**±**1.6 (13-18) | 52.6 | CAARMS / PANSS, CAARMS | CAARMS anhedonia measure showed significant correlations with negative symptoms (p<0.001). |
| Pitzianti 2019^124^ | Italy | Cross-sectional, (3) | 25 | 14.8**±**2 (7-18) | 68 | SIPS/SOPS / SIPS/SOPS | A significant correlation between total speed of timed activities and negative symptoms in CHR-P individuals (p=0.038). No correlation between overflow movements (p=0.718) and dysrhythmia (p=0.221) with negative symptoms was found. |
| Quijada 2010^125^ | Spain | Cross-sectional, (5) | 20 | 15.8 (12-20) | 60 | SIPS/SOPS, PANSS/ SIPS/SOPS, PANSS | 80% CHR-P experienced a reduction of motivation and poor work and school performance, 70% experienced a decrease in the ability to maintain or start social relationships and 55% social withdrawal. |
| Quijada 2012^126^ | Spain | Longitudinal, (4) | 31 | 15.7**±**3.1 (12-25) | 74 | SIPS/SOPS, PANSS/ SIPS/SOPS, PANSS | 71% improved in negative symptoms. No correlations between attachment (secure, preoccupied, fearful, dismissing) and negative symptoms were found (all p>0.05). |
| Rodriguez-Pascual 2021^127^ | Spain | Cross-sectional, (6) | 89 | 15.1**±**1.8 (8-17) | 40.7 | SIPS/SOPS/ PANSS | Significant differences in the presence of negative symptoms found between those with and without MDD (90.3% vs 68.2%, p=0.021). A correlation between negative symptoms and depressive symptoms was found (r=0.533, p<0.001). |
| Salazar de Pablo 2020^128^ | United States | Cross-sectional, (5) | 65 | 15.5**±**1.3 (12-17) | 24.6 | SIPS/SOPS, DSM-5/ SIPS/SOPS | Negative symptoms were significantly correlated with lower current functioning (r=−0.17; p=0.031), lower lowest functioning in the past year (r=−0.20; p=0.014) and lower highest functioning reached in the past year (r=−0.19; p=0.022). Illness severity was associated with negative symptoms (r=−039, p>0.001). |
| Schneider 2019^129^ | Multi-country | Longitudinal, (4) | 30 | 15.7**±**4.7 (8-33) | N.a. | SIPS/SOPS/ SIPS/SOPS | 66.7% CHR-P individuals had at least one negative symptoms. |
| Shetty 2021^130^ | Australia | Cross-sectional, (6) | 81 | 17.7**±**3.1 (15-29) | 38.3 | CAARMS/ SANS | Greater preference for eveningness was significantly associated with increased negative symptoms (p=0.02). |
| Spada 2016^131^ | Italy | Longitudinal, (5) | 22 | 16.1**±**1.2 (12-18) | 54.5 | CAARMS/ CAARMS | 77.3% CHR-P individuals had negative symptoms. 100% CHR-P individuals who transitioned to psychosis had negative symptoms (26.7 % of the total sample after a year). |
| White 2008^132^ | USA | Longitudinal, (4) | 68 | 17.3**±**3.8 (12-35) | 72 | SIPS/SOPS, SCID-DSM-IV/ SIPS/SOPS | Males were rated as having more severe negative symptoms than females at baseline, 6 months and 12 months follow-up (p<0.05). There was also a significant relationship between average negative symptoms and average global functioning (r=−0.75, p<0.001). |
| Zhang 2021^133^ | China | Longitudinal, (6) | 244 | 15.8**±**1.3 | 45.9 | SIPS/SOPS/ SIPS/SOPS | Negative symptoms were more severe in adolescents than adults (p<0.001). The conversion outcome was best predicted by negative symptoms compared to other clinical variables (p=0.006, d=0.46). |
|  |  |  |  |  |  |  |  |
| Akouri-Shan 2021^134^ | USA | Cross-sectional, (3) | 33 | 17.8**±**3.4 (12-23) | 40 | SIPS/SOPS/ SIPS/SOPS | Salience Attribution Test, including adaptive salience attribution did not correlate with negative symptoms (p> 0.05). Greater negative symptoms correlated with poorer social (r=0.7) and role functioning (r=0.5). |
| Mensi 2021^135^ | Italy | Longitudinal, (5) | 110 EOP | 15.5 (12-17) | 37.3 | DSM-5 | 74.3% of DSM-5 APS individuals reported negative symptoms. |
|  |  |  | 31 CHR-P | 14.5 (12-17) | 48.4 | CAARMS | 93.5% of EOP individuals reported negative symptoms. |
| Pihet 2013^136^ | Switzerland | Longitudinal, (4) | 28 | 15.6**±**1.3 (13-17) | 68.4 | DSM-IV, SIPS/ SOPS/ PANSS | Individuals who presented more severe negative symptoms at baseline had a significantly lower average treatment motivation (p<0.05). |
| Thomson 2019^137^ | UK | Longitudinal, (5) | 141 | 16.1**±**1.3 (13-18) | 56.7 | N.a./ PANSS | Including both CHR-P and EOP, negative symptoms improved at 12 months follow-up (p=0.002, d=0.46). Duration of untreated illness did not predict negative symptoms (p=0.07). Early negative symptoms predicter negative symptoms at 12 months follow up (r=0.62). |
| APS: attenuated psychosis syndrome CAARMS: Comprehensive Assessment of AT Risk Mental States; CBT: Cognitive Behavioural Therapy; CHR-P: Clinical High-Risk for Psychosis; DSM: Diagnostic and Statistical Manual of Mental Disorders; EOP: early onset psychosis ICD: International Classification of Diseases; IQ: Intelligence quotient; MDD: major depressive Disiroder; N.a. no available; PANSS: Positive and Negative Syndrome Scale; RCT: Randomised Clinical Trial; SANS: Scale for the Assessment of Negative Symptoms; RoB: risk of bias tool; SCID: Structured Clinical Interview for DSM Disorders; SIPS Structured Interview of Psychosis-risk Syndromes; SOPS Scale of Prodromal Symptoms; TAU: treatment as usual. | | | | | | | |

**eTable9. Publication bias: Egger’s test.**

|  | Intercept | SE | Z value | df | P value |
| --- | --- | --- | --- | --- | --- |
| Total | -1.66 | 1.953 | -0.85 | 19 | 0.3949 |
| EOP | -1.49 | 2.673 | -0.56 | 14 | 0.5780 |
| CHR-P | -2.65 | 1.393 | -1.90 | 5 | 0.0571 |

Df: degrees of freedom; SE: standard error.

**eFig1. Publication bias: funnel plot EOP and CHR-P.**

EOP: Early-onset psychosis; CHR-P: Clinical High-Risk for Psychosis.

**eFig2. Publication bias: funnel plot EOP.**

EOP: Early-onset psychosis.

**eFig3. Publication bias: funnel plot CHR-P.**

CHR-P: Clinical High-Risk for Psychosis.

**eMethods1. Search terms.**

We performed a multi-step literature search using the following keywords: (“child*” OR “adolesc*” OR “paediatric” OR “pediatric” OR “teen” OR “early-onset” OR “EOP”) AND (“psychosis” OR “schizophrenia” OR “schizoaffective” OR “prodrom*” OR “ultra-high risk” OR “clinical high risk” OR “clinical high-risk” OR “attenuat*” OR “APS” OR “high risk” OR “high-risk” OR “BLIPS” OR “brief limited” OR “brief intermittent” OR “genetic high risk” OR “genetic high-risk” OR “GRD” OR “at risk mental state” OR “at-risk mental state” OR “risk of progression” OR “progression to first-episode” OR “basic symptoms”) AND “negative symptoms”.

**eMethods2. Summary of included variables**

Summary of variables extracted included the following information: author and year of publication, country, study design (original or abstract; cross-sectional, longitudinal, randomised clinical trial, other intervention trial), study quality, sample size, age (mean age, SD and range), sex (% of males), diagnostic assessment/ instrument used, method used to assess negative symptoms, primary diagnosis (including % with schizophrenia), % individuals with negative symptoms, treatments received (including % antipsychotics), and key diagnostic, prognostic and therapeutic findings.

**eMethods3. Risk of bias (quality) assessment with Cochrane Risk of Bias tool for randomized clinical trials**

The Cochrane Risk of Bias tool^138^ was used to assess and classify the risk of bias in each of the included studies.

A judgement was made about whether each study had a high, low or unclear risk of bias in each of the following six domains: random sequence generation, allocation concealment, blinding of participants and study personnel, blinding of outcome assessments, incomplete outcome data, and selective outcome reporting.

The overall risk of bias was classified as low if none of the above domains was rated as high risk and three or less were rated as unclear risk. It was classified as unclear risk of bias if one domain was rated as high risk, or none rated as high risk but four or more rated as unclear risk. All other studies were classified as having a high risk of bias^139^.

**eResults1. Diagnostic categories and subgroups and negative symptoms in EOP.**

Negative symptoms seem to be more frequent in EOP individuals with a diagnosis of schizophrenia than with other psychosis in hospitalized individuals (p=0.04).^24^ Individuals with stable schizophrenia-spectrum disorder in the community seem to also have more severe negative symptoms than those with stable affective psychotic disorders (p<0.01).^39^ Further, negative symptoms were more severe in EOP with non-affective psychosis than affective psychosis (p=0.045)^128^ according to one of the studies. Specifically, in another study, individuals with schizophrenia (72%) and schizoaffective disorder (43%) had more negative symptoms at baseline than those with bipolar disorder (21%) or psychosis-NOS (9%).^53^ More severe negative symptoms were found in schizophrenia than in acute and transient psychotic disorders (p=0.006).^82^ Within schizophrenia, a trend toward higher negative symptom scores was found in the undifferentiated schizophrenia group compared to the paranoid schizophrenia group (p=0.05).^8^ In regards to other EOP subgroups, brief psychotic disorder was associated with lower maximum levels of negative symptoms compared to psychosis NOS (p=0.02).^17^

**eResults2. Neuroanatomical and neuroimaging and other neurobiological non-cognitive findings and negative symptoms in EOP.**

Altogether, 15 (15%) studies focused primarily on neuroanatomical and neuroimaging findings. A correlation between negative symptoms and smaller total cerebral volume was found (r=-0.65, p<0.001),^5^ although not consistently.^52^ Significant negative correlations between the bilateral hippocampal volumes and total negative symptoms (p<0.025) were also found.^23^ Grey matter volume of the hippocampus was negatively correlated with negative symptoms in EOP (r=-0.474, p=0.001).^99^ Negative symptoms were negatively correlated with reduced fiber counts through the frontal white matter (r=-0.39, p=0.043).^97^ EOP individuals with more predominant negative symptoms had more abnormalities in the superior temporal gyri-inferior frontal gyri network (p<0.05).^101^ Voxel-wise amplitude of low-frequency (0.01–0.08 Hz) fluctuations in the precuneus had a significant negative correlation with PANSS negative symptom scores (r=-0.44, p=0.0067).^103^ Whole-brain correlation analysis found that the degree of centrality in the right superior parietal lobe was positively correlated with PANSS-negative symptom scores (r=0.79).^104^ The precuneus with reduced global functional connectivity density was significantly negatively correlated with negative symptoms in the slow-4 band (p<0.05).^96^ There was a significant correlation between decreased IL-10 and increased IL-4 levels with increased negative symptoms (r=-0.65, p=0.02 and r=0.67, p=0.02, respectively).^89^ The loss of grey matter volume did not correlate with negative symptoms (p<0.05).^91^ Triglyceride levels were significantly higher in the severe negative group than in the mild negative individual group (p=0.032).^98^

A negative correlation was found between negative symptoms and the abnormal dynamic amplitude of low-frequency fluctuations (dALFF) variability of the right middle temporal gyrus (r=-0.2723, p=0.0207).^50^ The risk allele A of ZNF804A was associated with greater severity of negative symptoms in EOP (p<0.05).^6^ The Val/Val group had a higher negative symptom score compared to the Val/Met group (p=0.018, Cohen’s d=0.55).^42^ No association found between negative symptoms and sleep spindle density (p=0.07).^32^

**References**

1. McGlashan T, Walsh B, Woods S. The Psychosis-Risk Syndrome: Handbook for Diagnosis and Follow-Up2010.

2. Fusar-Poli P, Cappucciati M, Rutigliano G, Lee TY, Beverly Q, Bonoldi I, et al. Towards a Standard Psychometric Diagnostic Interview for Subjects at Ultra High Risk of Psychosis: CAARMS versus SIPS. Psychiatry J. 2016; 2016: 7146341.

3. Schultze-Lutter F, Theodoridou A. The concept of basic symptoms: its scientific and clinical relevance. World Psychiatry. 2017; 16(1): 104-5.

4. Vaquerizo-Serrano J, Salazar de Pablo G, Singh J, Santosh P. Autism Spectrum Disorder and Clinical High Risk for Psychosis: A Systematic Review and Meta-analysis. J Autism Dev Disord. 2022; 52(4): 1568-86.

5. Alaghband-Rad J, Hamburger SD, Giedd JN, Frazier JA, Rapoport JL. Childhood-onset schizophrenia: biological markers in relation to clinical characteristics. The American journal of psychiatry. 1997; 154(1): 64-8.

6. Alfimova MV, Nikitina SG, Lezheiko TV, Simashkova NV, Golimbet VE. Impact on the Risk and Severity of Childhood Onset Schizophrenia of Schizophrenia Risk Genetic Variants at the DRD2 and ZNF804A Loci. Child Psychiatry & Human Development. 2021.

7. Arango C, Rapado-Castro M, Reig S, Castro-Fornieles J, Gonzalez-Pinto A, Otero S, et al. Progressive Brain Changes in Children and Adolescents With First-Episode Psychosis. Archives of General Psychiatry. 2012; 69(1): 16-26.

8. Bellgrove MA, Chambers CD, Vance A, Hall N, Karamitsios M, Bradshaw JL. Lateralized deficit of response inhibition in early-onset schizophrenia. Psychological Medicine. 2006; 36(4): 495-505.

9. Bunk D, Eggers C, Klapal M. Symptom dimensions in the course of childhood-onset schizophrenia. European child & adolescent psychiatry. 1999; 8 Suppl 1: I29-35.

10. Burton CZ, Tso IF, Carrion RE, Niendam T, Adelsheim S, Auther AM, et al. Baseline psychopathology and relationship to longitudinal functional outcome in attenuated and early first episode psychosis. Schizophrenia Research. 2019; 212: 157-62.

11. Calderon-Mediavilla M, Vila-Badia R, Dolz M, Butjosa A, Barajas A, Del Cacho N, et al. Depressive symptoms and their relationship with negative and other psychotic symptoms in early onset psychosis. European Child & Adolescent Psychiatry. 2021; 30(9): 1383-90.

12. Calvo A, Moreno M, Ruiz-Sancho A, Rapado-Castro M, Moreno C, Sanchez-Gutierrez T, et al. Intervention for Adolescents With Early-Onset Psychosis and Their Families: A Randomized Controlled Trial. Journal of the American Academy of Child and Adolescent Psychiatry. 2014; 53(6): 688-96.

13. Calvo A, Moreno M, Ruiz-Sancho A, Rapado-Castro M, Moreno C, Sanchez-Gutierrez T, et al. Psychoeducational Group Intervention for Adolescents With Psychosis and Their Families: A Two-Year Follow-Up. Journal of the American Academy of Child and Adolescent Psychiatry. 2015; 54(12): 984-90.

14. Cheng X, Zhang H, Zhang J, Xu P, Jin P, Fang H, et al. Comparison of clinical characteristics and treatment efficacy in childhood-onset schizophrenia and adolescent-onset schizophrenia in mainland China: A retrospective study. Early Intervention in Psychiatry. 2021; 15(6): 1721-9.

15. Cohen E, Sereni N, Kaplan O, Weizman A, Kikinzon L, Weiner I, et al. The relation between latent inhibition and symptom-types in young schizophrenics. Behavioural Brain Research. 2004; 149(2): 113-22.

16. Corcoran C, Whitaker A, Coleman E, Fried J, Feldman J, Goudsmit N, et al. Olfactory deficits, cognition and negative symptoms in early onset psychosis. Schizophrenia Research. 2005; 80(2-3): 283-93.

17. Correll CU, Lencz T, Smith CW, Auther AM, Nakayama EY, Hovey L, et al. Prospective study of adolescents with subsyndromal psychosis: Characteristics and outcome. Journal of Child and Adolescent Psychopharmacology. 2005; 15(3): 418-33.

18. Correll CU, Smith CW, Auther AM, McLaughlin D, Shah M, Foley C, et al. Predictors of Remission, Schizophrenia, and Bipolar Disorder in Adolescents with Brief Psychotic Disorder or Psychotic Disorder Not Otherwise Specified Considered At Very High Risk for Schizophrenia. Journal of Child and Adolescent Psychopharmacology. 2008; 18(5): 475-90.

19. Correll CU, Tocco M, Hsu J, Goldman R, Pikalov A. Short-term efficacy and safety of lurasidone versus placebo in antipsychotic-naive versus previously treated adolescents with an acute exacerbation of schizophrenia. European Psychiatry. 2022; 65(1).

20. Craddock KES, Zhou X, Liu S, Gochman P, Dickinson D, Rapoport JL. Symptom dimensions and subgroups in childhood-onset schizophrenia. Schizophr Res. 2018; 197: 71-7.

21. DeVylder JE, Gearing RE. Declining social support in adolescents prior to first episode psychosis: Associations with negative and affective symptoms. Psychiatry Research. 2013; 210(1): 50-4.

22. Downs J, Dean H, Lechler S, Sears N, Patel R, Shetty H, et al. Negative Symptoms in Early-Onset Psychosis and Their Association With Antipsychotic Treatment Failure. Schizophrenia Bulletin. 2019; 45(1): 69-79.

23. Duan X, He C, Ou J, Wang R, Xiao J, Li L, et al. Reduced Hippocampal Volume and Its Relationship With Verbal Memory and Negative Symptoms in Treatment-Naive First-Episode Adolescent-Onset Schizophrenia. Schizophrenia Bulletin. 2021; 47(1): 64-74.

24. Fields JH, Grochowski S, Lindenmayer JP, Kay SR, Grosz D, Hyman RB, et al. Assessing positive and negative symptoms in children and adolescents. American Journal of Psychiatry. 1994; 151(2): 249-53.

25. Findling RL, McNamara NK, Youngstrom EA, Branicky LA, Demeter CA, Schulz SC. A prospective, open-label trial of olanzapine in adolescents with schizophrenia. Journal of the American Academy of Child and Adolescent Psychiatry. 2003; 42(2): 170-5.

26. Fleischhaker C, Schulz E, Tepper K, Martin M, Hennighausen K, Remschmidt H. Long-term course of adolescent schizophrenia. Schizophrenia Bulletin. 2005; 31(3): 769-80.

27. Fraguas D, Merchan-Naranjo J, del Rey-Mejias A, Castro-Fornieles J, Gonzalez-Pinto A, Rapado-Castro M, et al. A longitudinal study on the relationship between duration of untreated psychosis and executive function in early-onset first-episode psychosis. Schizophrenia Research. 2014; 158(1-3): 126-33.

28. Garcia Traverso M, Fraguas D, Arango C, Castro-Fornieles J, Gonzalez-Pinto A, Parellada M, et al. Longitudinal assessment of negative symptoms in early onset first episode psychosis. European Neuropsychopharmacology. 2019; 29: S429-S30.

29. Garcia-Amador M, Janssen J, Reig S, Parellada M, Moreno D, Moreno C, et al. Cortical thickness is associated with clinical improvement of negative symptoms in male adolescents with early-onset first-episode psychosis. Schizophrenia Research. 2010; 117(2-3): 227-8.

30. Garcia-Amador M, Joost J, Reig S, Parellada M, Moreno D, Moreno C, et al. Frontal cortical thickness is associated with clinical improvement of negative symptoms in male adolescents with early-onset first-episode psychosis. Schizophrenia Research. 2010; 117(2-3): 225-6.

31. Geon HB, 노봉근, 윤도준, 김용희, 염태호. Comparison of Clinical Characteristics between Inpatients with Early & Late-Onset Schizophrenia. Journal of the Korean Society of Biological Therapies in Psychiatry. 2002; 8(2): 242-53.

32. Gerstenberg M, Hauser M, Al-Jadiri A, Sheridan EM, Kishimoto T, Borenstein Y, et al. Frequency and Correlates of DSM-5 Attenuated Psychosis Syndrome in a Sample of Adolescent Inpatients With Nonpsychotic Psychiatric Disorders. Journal of Clinical Psychiatry. 2015; 76(11): 1449-58.

33. Gothelf D, Apter A, Reidman J, Brand-Gothelf A, Bloch Y, Gal G, et al. Olanzapine, risperidone and haloperidol in the treatment of adolescent patients with schizophrenia. Journal of Neural Transmission. 2003; 110(5): 545-60.

34. Hadjez J, Stein D, Gabbay U, Bruckner J, Meged S, Barak Y, et al. Dream content of schizophrenic, nonschizophrenic mentally ill, and community control adolescents. Adolescence. 2003; 38(150): 331-42.

35. Hassan GAM, Taha GRA. Long term functioning in early onset psychosis: Two years prospective follow-up study. Behavioral and Brain Functions. 2011; 7.

36. Hemmerle MJ, Roepcke B, Eggers C, Oades RD. Evaluation of a two-year intensive outpatient care programme for adolescents with schizophrenia. Zeitschrift Fur Kinder-Und Jugendpsychiatrie Und Psychotherapie. 2010; 38(5): 361-9.

37. Hintze B, Borkowska A. Associations Between Cognitive Function, Schizophrenic Symptoms, and Functional Outcome in Early-onset Schizophrenia With and Without a Familial Burden of Psychosis. Israel Journal of Psychiatry and Related Sciences. 2015; 52(3): 6-13.

38. Hollis C. Developmental precursors of child- and adolescent-onset schizophrenia and affective psychoses: diagnostic specificity and continuity with symptom dimensions. British Journal of Psychiatry. 2003; 182: 37-44.

39. Jarbin H, von Knorring AL. Diagnostic stability in adolescent onset psychotic disorders. European Child & Adolescent Psychiatry. 2003; 12(1): 15-22.

40. Jarbin H, von Knorring AL. Suicide and suicide attempts in adolescent-onset psychotic disorders. Nordic Journal of Psychiatry. 2004; 58(2): 115-23.

41. Kafali HY, Bildik T, Bora E, Yuncu Z, Erermis HS. Distinguishing prodromal stage of bipolar disorder and early onset schizophrenia spectrum disorders during adolescence. Psychiatry Research. 2019; 275: 315-25.

42. Karacetin G, Bayoglu B, Soylemez TE, Topal M, Koc EB, Tekden M, et al. BDNF Val66Met polymorphism is associated with negative symptoms in early-onset schizophrenia spectrum and other psychotic disorders. European Journal of Psychiatry. 2022; 36(1): 26-34.

43. Karakus OB, Ermis C, Tuncturk M, Yuksel AS, Alarslan S, Saglam Y, et al. Identifying clinical and psychological correlates of persistent negative symptoms in early-onset psychotic disorders. Clinical Child Psychology and Psychiatry. 2022.

44. Kemp DE, Correll CU, Tohen M, DelBello MP, Ganocy SJ, Findling RL, et al. Associations Among Obesity, Acute Weight Gain, and Response to Treatment with Olanzapine in Adolescent Schizophrenia. Journal of Child and Adolescent Psychopharmacology. 2013; 23(8): 522-30.

45. Kim Y, Cho S-C, Shin M-S, Kim J-W, Choi S-C, Kim B-N. Aripiprazole in the Treatment of Early-Onset Schizophrenia Spectrum Disorder: A Case Series in Korean Children and Adolescents. Current Therapeutic Research-Clinical and Experimental. 2009; 70(2): 173-83.

46. Pyung-Soon Kim MD, Jae-Ho Shin MD, 이창화. Comparison of Clinical Characteristics between Patients with Early-Onset and Adult-Onset Schizophrenia : A Retrospective Study. Journal of Korean Society of Biological Psychiatry. 2013; 20(4): 179-86.

47. Kumra S, Frazier JA, Jacobsen LK, McKenna K, Gordon CT, Lenane MC, et al. Childhood-onset schizophrenia - A double-blind clozapine-haloperidol comparison. Archives of General Psychiatry. 1996; 53(12): 1090-7.

48. Kumra S, Jacobsen LK, Lenane M, Karp BI, Frazier JA, Smith AK, et al. Childhood-onset schizophrenia: An open-label study of olanzapine in adolescents. Journal of the American Academy of Child and Adolescent Psychiatry. 1998; 37(4): 377-85.

49. Kumra S, Kranzler H, Gerbino-Rosen G, Kester HM, DeThomas C, Kafantaris V, et al. Clozapine and "high-dose" olanzapine in refractory early-onset schizophrenia: A 12-week randomized and double-blind comparison. Biological Psychiatry. 2008; 63(5): 524-9.

50. Li Q, Cao X, Liu S, Li Z, Wang Y, Cheng L, et al. Dynamic Alterations of Amplitude of Low-Frequency Fluctuations in Patients With Drug-Naive First-Episode Early Onset Schizophrenia. Frontiers in Neuroscience. 2020; 14.

51. Maki PH, Miettunen J, Kaakinen M, Moilanen I, Taanila A, Jones PB, et al. Negative symptoms precede the onset of first episode psychosis in a prospective general population sample of adolescents. European Psychiatry. 2008; 23: S129-S.

52. Matsumoto H, Simmons A, Williams S, Pipe R, Murray R, Frangou S. Structural magnetic imaging of the hippocampus in early onset schizophrenia. Biological Psychiatry. 2001; 49(10): 824-31.

53. McClellan J, McCurry C. Early onset psychotic disorders: Diagnostic stability and clinical characteristics. European Child & Adolescent Psychiatry. 1999; 8: 13-9.

54. McClellan J, McCurry C, Snell J, DuBose A. Early-onset psychotic disorders: Course and outcome over a 2-year period. Journal of the American Academy of Child and Adolescent Psychiatry. 1999; 38(11): 1380-8.

55. McClellan J, McCurry C, Speltz ML, Jones K. Symptom factors in early-onset psychotic disorders. Journal of the American Academy of Child and Adolescent Psychiatry. 2002; 41(7).

56. McClellan J, Breiger D, McCurry C, Hlastala SA. Premorbid functioning in early-onset psychotic disorders. Journal of the American Academy of Child and Adolescent Psychiatry. 2003; 42(6): 666-72.

57. McConville BJ, Arvanitis LA, Thyrum PT, Yeh C, Wilkinson LA, Chaney RO, et al. Pharmacokinetics, tolerability, and clinical effectiveness of quetiapine fumarate: An open-label trial in adolescents with psychotic disorders. Journal of Clinical Psychiatry. 2000; 61(4): 252-60.

58. McConville B, Carrero L, Sweitzer D, Potter L, Chaney R, Foster K, et al. Long-term safety, tolerability, and clinical efficacy of quetiapine in adolescents: An open-label extension trial. Journal of Child and Adolescent Psychopharmacology. 2003; 13(1): 75-82.

59. Meng H, Schimmelmann BG, Mohler B, Lambert M, Branik E, Koch E, et al. Pretreatment social functioning predicts 1-year outcome in early onset psychosis. Acta Psychiatrica Scandinavica. 2006; 114(4): 249-56.

60. Merchan-Naranjo J, Fraguas D, De Castro MJ, Parellada M, Moreno D, Ruiz-Sancho A, et al. Negative symptoms predict functional outcome of early-onset psychosis. European Psychiatry. 2007; 22: S125-S.

61. Morch-Johnsen L, Smelror RE, Andreou D, Barth C, Johannessen C, Wedervang-Resell K, et al. Negative Symptom Domains Are Associated With Verbal Learning in Adolescents With Early Onset Psychosis. Frontiers in Psychiatry. 2022; 12.

62. Mozes T, Ebert T, Michal S-E, Spivak B, Weizman A. An open-label randomized comparison of olanzapine versus risperidone in the treatment of childhood-onset schizophrenia. Journal of Child and Adolescent Psychopharmacology. 2006; 16(4): 393-403.

63. Mueller H, Kommescher M, Guettgemanns J, Wessels H, Walger P, Lehmkuhl G, et al. Cognitive behavioral therapy in adolescents with early-onset psychosis: a randomized controlled pilot study. European Child & Adolescent Psychiatry. 2020; 29(7): 1011-22.

64. Nechmad A, Ratzoni G, Poyurovsky M, Meged S, Avidan G, Fuchs C, et al. Obsessive-compulsive disorder in adolescent schizophrenia patients. Am J Psychiatry. 2003; 160(5): 1002-4.

65. Oades RD, Wild-Wall N, Juran SA, Sachsse J, Oknina LB, Roepcke B. Auditory change detection in schizophrenia: sources of activity, related neuropsychological function and symptoms in patients with a first episode in adolescence, and patients 14 years after an adolescent illness-onset. Bmc Psychiatry. 2006; 6.

66. Paillere-Martinot ML, Aubin F, Martinot JL, Colin B. A prognostic study of clinical dimensions in adolescent-onset psychoses. Schizophrenia Bulletin. 2000; 26(4): 789-99.

67. Pandina G, Nuamah I, Petersen T, Singh J, Savitz A, Hough D. Cognitive functioning in adolescents with schizophrenia treated with paliperidone extended-release: 6-Month exploratory analysis from an open-label, single-arm safety study. Schizophrenia Research-Cognition. 2020; 20.

68. Parellada MJ, Moreno D, Ruiz-Sancho A, Medina O, Arango C. Open-label randomized trial comparing the efficacy and tolerability of quetiapine and olanzapine in first episode psychosis in adolescents. European Neuropsychopharmacology. 2006; 16: S377-S8.

69. Parellada M, Castro-Fornieles J, Gonzalez-Pinto A, Pina-Camacho L, Moreno D, Rapado-Castro M, et al. Predictors of Functional and Clinical Outcome in Early-Onset First-Episode Psychosis: The Child and Adolescent First Episode of Psychosis (CAFEPS) Study. Journal of Clinical Psychiatry. 2015; 76(11): E1441-+.

70. Paruk S, Jhazbhay K, Singh K, Sartorius B, Burns JK. The clinical impact of a positive family history of psychosis or mental illness in psychotic and non-psychotic mentally ill adolescents. Journal of Child and Adolescent Mental Health. 2017; 29(3): 219-29.

71. Pencer A, Addington J, Addington D. Outcome of a first episode of psychosis in adolescence: a 2-year follow-up. Psychiatry Research. 2005; 133(1): 35-43.

72. Perez-Garza R, Victoria-Figueroa G, Elena Ulloa-Flores R. Sex Differences in Severity, Social Functioning, Adherence to Treatment, and Cognition of Adolescents with Schizophrenia. Schizophrenia Research and Treatment. 2016; 2016.

73. Petruzzelli MG, Margari L, Bosco A, Craig F, Palumbi R, Margari F. Early onset first episode psychosis: dimensional structure of symptoms, clinical subtypes and related neurodevelopmental markers. Eur Child Adolesc Psychiatry. 2018; 27(2): 171-9.

74. Poyurovsky M, Faragian S, Shabeta A, Kosov A. Comparison of clinical characteristics, co-morbidity and pharmacotherapy in adolescent schizophrenia patients with and without obsessive-compulsive disorder. Psychiatry Research. 2008; 159(1-2): 133-9.

75. Puig O, Penades R, Baeza I, Sanchez-Gistau V, De la Serna E, Fonrodona L, et al. Processing speed and executive functions predict real-world everyday living skills in adolescents with early-onset schizophrenia. European Child & Adolescent Psychiatry. 2012; 21(6): 315-26.

76. Ramanathan S, Miewald J, Montrose D, Keshavan MS. Can age at sexual maturity act as a predictive biomarker for prodromal negative symptoms? Schizophrenia Research. 2015; 164(1-3): 35-9.

77. Rapado-Castro M, Soutullo C, Fraguas D, Arango C, Paya B, Castro-Fornieles J, et al. Predominance of Symptoms Over Time in Early-Onset Psychosis: A Principal Component Factor Analysis of the Positive and Negative Syndrome Scale. Journal of Clinical Psychiatry. 2010; 71(3): 327-37.

78. Rapado-Castro M, Moreno C, Ruiz-Sancho A, Camino F, Arango C, Mayoral M. Role of Executive Function in Response to a Problem Solving Based Psychoeducational Intervention in Adolescents with Psychosis: The PIENSA Trial Revisited. Journal of Clinical Medicine. 2019; 8(12).

79. Reddy YCJ, Srinath S, Sathyanarayana V, Girimaji S, Seshadri S. Clinical profile of early onset schizophrenia: A review of 43 cases. Nimhans Journal. 1996; 14(2): 93-8.

80. Reig S, Parellada M, Castro-Fornieles J, Janssen J, Moreno D, Baeza I, et al. Multicenter Study of Brain Volume Abnormalities in Children and Adolescent-Onset Psychosis. Schizophrenia Bulletin. 2011; 37(6): 1270-80.

81. Remberk B, Namyslowska I, Rybakowski F. Cognition and communication dysfunctions in early-onset schizophrenia: Effect of risperidone. Progress in Neuro-Psychopharmacology & Biological Psychiatry. 2012; 39(2): 348-54.

82. Remberk B, Bogumil B, Bronowska Z, Namyslowska I, Potocki P. Retrospective analysis of psychopathological presentation of psychotic episodes in adolescent inpatients. Psychiatria Polska. 2012; 46(2): 177-88.

83. Remberk B, Bazynska AK, Bronowska Z, Potocki P, Krempa-Kowalewska A, Niwinski P, et al. Which Aspects of Long-Term Outcome Are Predicted by Positive and Negative Symptoms in Early-Onset Psychosis? An Exploratory Eight-Year Follow-Up Study. Psychopathology. 2015; 48(1): 47-55.

84. Ross RG, Novins D, Farley GK, Adler LE. A 1-year open-label trial of olanzapine in school-age children with schizophrenia. Journal of Child and Adolescent Psychopharmacology. 2003; 13(3): 301-9.

85. Salazar de Pablo G, Moreno D, Gonzalez-Pinto A, Paya B, Castro-Fonieles J, Baeza I, et al. Affective symptom dimensions in early-onset psychosis over time: a principal component factor analysis of the Young Mania Rating Scale and the Hamilton Depression Rating Scale. Eur Child Adolesc Psychiatry. 2021.

86. Savitz AJ, Lane R, Nuamah I, Gopal S, Hough D. Efficacy and Safety of Paliperidone Extended Release in Adolescents With Schizophrenia: A Randomized, Double-Blind Study. Journal of the American Academy of Child and Adolescent Psychiatry. 2015; 54(2): 126-37.

87. Schwartz-Stav O, Apter A, Zalsman G. Depression, suicidal behavior and insight in adolescents with schizophrenia. Eur Child Adolesc Psychiatry. 2006; 15(6): 352-9.

88. Shaw P, Sporn A, Gogtay N, Overman GP, Greenstein D, Gochman P, et al. Childhood-onset schizophrenia - A double-blind, randomized clozapine-olanzapine comparison. Archives of General Psychiatry. 2006; 63(7): 721-30.

89. Şimşek Ş, Yıldırım V, Çim A, Kaya S. Serum IL-4 and IL-10 Levels Correlate with the Symptoms of the Drug-Naive Adolescents with First Episode, Early Onset Schizophrenia. J Child Adolesc Psychopharmacol. 2016; 26(8): 721-6.

90. Slosarczyk M, Slosarczyk K, Furgal M. A 45-year follow-up study of adolescent schizophrenia. Part I: Premorbid values of psychological and relational indicators in the context of illness course and long-term social functioning of patients. Psychiatria Polska. 2022; 56(1): 19-34.

91. Tang J, Liao Y, Zhou B, Tan C, Liu W, Wang D, et al. Decrease in Temporal Gyrus Gray Matter Volume in First-Episode, Early Onset Schizophrenia: An MRI Study. Plos One. 2012; 7(7).

92. Thaden E, Rhinewine JP, Lencz T, Kester H, Cervellione KL, Henderson I, et al. Early-onset schizophrenia is associated with impaired adolescent development of attentional capacity using the identical pairs continuous performance test. Schizophrenia Research. 2006; 81(2-3): 157-66.

93. Thakur A, Jagadheesan K, Sinha VK. Psychopathological dimensions in childhood and adolescent psychoses: A confirmatory factor analytical study. Psychopathology. 2003; 36(4): 190-4.

94. Vines L, Bridgwater M, Bachman P, Hayes R, Catalano S, Jalbrzikowski M. Elevated emotion reactivity and emotion regulation in individuals at clinical high risk for developing psychosis and those diagnosed with a psychotic disorder. Early Intervention in Psychiatry. 2022; 16(7): 724-35.

95. Vyas NS, Hadjulis M, Vourdas A, Byrne P, Frangou S. The Maudsley early onset schizophrenia study - Predictors of psychosocial outcome at 4-year follow-up. European Child & Adolescent Psychiatry. 2007; 16(7): 465-70.

96. Wang X, Zhang Y, Long Z, Zheng J, Han S, Wang Y, et al. Frequency-specific alteration of functional connectivity density in antipsychotic-naive adolescents with early-onset schizophrenia. J Psychiatr Res. 2017; 95: 68-75.

97. Wang X, Lu F, Duan X, Han S, Guo X, Yang M, et al. Frontal white matter abnormalities reveal the pathological basis underlying negative symptoms in antipsychotic-naive, first-episode patients with adolescent-onset schizophrenia: Evidence from multimodal brain imaging. Schizophrenia Research. 2020; 222: 258-66.

98. Wedervang-Resell K, Friis S, Lonning V, Smelror RE, Johannessen C, Agartz I, et al. Lipid alterations in adolescents with early-onset psychosis may be independent of antipsychotic medication. Schizophrenia Research. 2020; 216: 295-301.

99. Wen D, Wang J, Yao G, Liu S, Li X, Li J, et al. Abnormality of subcortical volume and resting functional connectivity in adolescents with early-onset and prodromal schizophrenia. Journal of Psychiatric Research. 2021; 140: 282-8.

100. Wozniak JR, Block EE, White T, Jensen JB, Schulz SC. Clinical and neurocognitive course in early-onset psychosis: a longitudinal study of adolescents with schizophrenia-spectrum disorders. Early Intervention in Psychiatry. 2008; 2(3): 169-77.

101. Yang Z, Xu Y, Xu T, Hoy CW, Handwerker DA, Chen G, et al. Brain Network Informed Subject Community Detection In Early-Onset Schizophrenia. Scientific Reports. 2014; 4.

102. Zakowicz P, Skibinska M, Pawlak J. Disembodied Language in Early-Onset Schizophrenia. Frontiers in Psychiatry. 2022; 13.

103. Zheng J, Zhang Y, Guo X, Duan X, Zhang J, Zhao J, et al. Disrupted amplitude of low-frequency fluctuations in antipsychotic-naïve adolescents with early-onset schizophrenia. Psychiatry Res Neuroimaging. 2016; 249: 20-6.

104. Zhou M, Zhuo L, Ji R, Gao Y, Yao H, Feng R, et al. Alterations in functional network centrality in first-episode drug-naive adolescent-onset schizophrenia. Brain Imaging and Behavior. 2022; 16(1): 316-23.

105. Addington J, Shah H, Liu L, Addington D. Reliability and validity of the Calgary Depression Scale for Schizophrenia (CDSS) in youth at clinical high risk for psychosis. Schizophrenia Research. 2014; 153(1-3): 64-7.

106. Amminger GP, Schafer MR, Papageorgiou K, Klier CM, Cotton SM, Harrigan SM, et al. Long-Chain omega-3 Fatty Acids for Indicated Prevention of Psychotic Disorders A Randomized, Placebo-Controlled Trial. Archives of General Psychiatry. 2010; 67(2): 146-54.

107. Amminger GP, Mechelli A, Rice S, Kim SW, Klier CM, McNamara RK, et al. Predictors of treatment response in young people at ultra-high risk for psychosis who received long-chain omega-3 fatty acids. Translational Psychiatry. 2015; 5.

108. Armando M, Girardi P, Vicari S, Menghini D, Digilio MC, Pontillo M, et al. Adolescents at ultra-high risk for psychosis with and without 22q11 deletion syndrome: A comparison of prodromal psychotic symptoms and general functioning. Schizophrenia Research. 2012; 139(1-3): 151-6.

109. Bartholomeusz CF, Whittle SL, Pilioussis E, Allott K, Rice S, Schaefer MR, et al. Relationship between amygdala volume and emotion recognition in adolescents at ultra-high risk for psychosis. Psychiatry Research-Neuroimaging. 2014; 224(3): 159-67.

110. Boldrini T, Lo Buglio G, Giovanardi G, Lingiardi V, Salcuni S. Defense mechanisms in adolescents at high risk of developing psychosis: an empirical investigation. Research in Psychotherapy-Psychopathology Process and Outcome. 2020; 23(1): 4-15.

111. Gerstenberg M, Furrer M, Tesler N, Franscini M, Walitza S, Huber R. Reduced sleep spindle density in adolescent patients with early-onset schizophrenia compared to major depressive disorder and healthy controls. Schizophrenia Research. 2020; 221: 20-8.

112. Giordano. Negative Symptom Domains in Children and Adolescents at Ultra-High Risk for

Psychosis: Association With Real-Life Functioning. 2022.

113. Lencz T, Smith CW, Auther A, Correll CU, Cornblatt B. Nonspecific and attenuated negative symptoms in patients at clinical high-risk for schizophrenia. Schizophr Res. 2004; 68(1): 37-48.

114. Lindgren M, Manninen M, Laajasalo T, Mustonen U, Kalska H, Suvisaari J, et al. The relationship between psychotic-like symptoms and neurocognitive performance in a general adolescent psychiatric sample. Schizophrenia Research. 2010; 123(1): 77-85.

115. Lindgren M, Kuvaja H, Jokela M, Therman S. Predictive validity of psychosis risk models when applied to adolescent psychiatric patients. Psychological Medicine. 2021: 1-12.

116. McGorry PD, Nelson B, Phillips LJ, Yuen HP, Francey SM, Thampi A, et al. Randomized Controlled Trial of Interventions for Young People at Ultra-High Risk of Psychosis: Twelve-Month Outcome. Journal of Clinical Psychiatry. 2013; 74(4): 349-56.

117. Miklowitz DJ, O'Brien MP, Schlosser DA, Addington J, Candan KA, Marshall C, et al. Family-focused treatment for adolescents and young adults at high risk for psychosis: results of a randomized trial. J Am Acad Child Adolesc Psychiatry. 2014; 53(8): 848-58.

118. Niendam TA, Bearden CE, Johnson JK, McKinley M, Loewy R, O'Brien M, et al. Neurocognitive performance and functional disability in the psychosis prodrome. Schizophrenia Research. 2006; 84(1): 100-11.

119. O'Brien MP, Gordon JL, Bearden CE, Lopez SR, Kopelowicz A, Cannon TD. Positive family environment predicts improvement in symptoms and social functioning among adolescents at imminent risk for onset of psychosis. Schizophrenia Research. 2006; 81(2-3): 269-75.

120. O'Brien MP, Zinberg JL, Bearden CE, Lopez SR, Kopelowicz A, Daley M, et al. Parent attitudes and parent adolescent interaction in families of youth at risk for psychosis and with recent-onset psychotic symptoms. Early Intervention in Psychiatry. 2008; 2(4): 268-76.

121. Pelizza L, Azzali S, Garlassi S, Paterlini F, Scazza I, Chiri LR, et al. Adolescents at ultra-high risk of psychosis in Italian neuropsychiatry services: prevalence, psychopathology and transition rate. European Child & Adolescent Psychiatry. 2018; 27(6): 725-37.

122. Pelizza L, Poletti M, Azzali S, Garlassi S, Scazza I, Paterlini F, et al. Subjective experience of social cognition in adolescents at ultra-high risk of psychosis: findings from a 24-month follow-up study. Eur Child Adolesc Psychiatry. 2020.

123. Pelizza L, Poletti M, Azzali S, Paterlini F, Garlassi S, Scazza I, et al. Anhedonia in adolescents at ultra-high risk (UHR) of psychosis: findings from a 1-year longitudinal study. Eur Arch Psychiatry Clin Neurosci. 2020; 270(3): 337-50.

124. Pitzianti M, Pontillo M, Vicari S, Armando M, Pasini A. 22q11 microdeletion syndrome and ultra-high risk for psychosis: The role of neurological soft signs as an independent marker of vulnerability for psychosis. Early Intervention in Psychiatry. 2019; 13(5): 1191-8.

125. Quijada Y, Tizon JL, Artigue J, Parra B. At-risk mental state (ARMS) detection in a community service center for early attention to psychosis in Barcelona. Early Intervention in Psychiatry. 2010; 4(3): 257-62.

126. Quijada Y, Tizon JL, Artigue J, Kwapil TR, Barrantes-Vidal N. Attachment style predicts 6-month improvement in psychoticism in persons with at-risk mental states for psychosis. Early Intervention in Psychiatry. 2012; 6(4): 442-9.

127. Rodriguez-Pascual M, Alvarez-Subiela X, Tor J, Pardo M, de la Serna E, Sugranyes G, et al. Major depressive disorder and attenuated negative symptoms in a child and adolescent sample with psychosis risk syndrome: the CAPRIS study. European Child & Adolescent Psychiatry. 2021.

128. Salazar de Pablo G, Guinart D, Cornblatt BA, Auther AM, Carrión RE, Carbon M, et al. DSM-5 Attenuated Psychosis Syndrome in Adolescents Hospitalized With Non-psychotic Psychiatric Disorders. Front Psychiatry. 2020; 11: 568982.

129. Schneider M, Armando M, Schultze-Lutter F, Pontillo M, Vicari S, Debbane M, et al. Prevalence, course and psychosis-predictive value of negative symptoms in 22q11.2 deletion syndrome. Schizophrenia Research. 2019; 206: 386-93.

130. Shetty JJ, Nicholas C, Nelson B, McGorry PD, Lavoie S, Markulev C, et al. Greater preference for eveningness is associated with negative symptoms in an ultra-high risk for psychosis sample. Early Intervention in Psychiatry. 2021; 15(6): 1793-8.

131. Spada G, Molteni S, Pistone C, Chiappedi M, McGuire P, Fusar-Poli P, et al. Identifying children and adolescents at ultra high risk of psychosis in Italian neuropsychiatry services: a feasibility study. European Child & Adolescent Psychiatry. 2016; 25(1): 91-106.

132. White RK, Niendam TA, Bearden CE, Zinberg J, O'Brien MP, Cannon TD. Gender differences in symptoms, functioning and social support in patients at ultra-high risk for developing a psychotic disorder. Schizophrenia Research. 2008; 104(1-3): 237-45.

133. Zhang T, Xu L, Chen Y, Wei Y, Tang X, Hu Y, et al. Conversion to psychosis in adolescents and adults: similar proportions, different predictors. Psychological Medicine. 2021; 51(12): 2003-11.

134. Akouri-Shan L, Schiffman J, Millman ZB, Demro C, Fitzgerald J, Rouhakhtar PJR, et al. Relations Among Anhedonia, Reinforcement Learning, and Global Functioning in Help-seeking Youth. Schizophrenia Bulletin. 2021; 47(6): 1534-43.

135. Mensi MM, Molteni S, Iorio M, Filosi E, Ballante E, Balottin U, et al. Prognostic Accuracy of DSM-5 Attenuated Psychosis Syndrome in Adolescents: Prospective Real-World 5-Year Cohort Study. Schizophrenia Bulletin. 2021; 47(6): 1663-73.

136. Pihet S, Passini CM, Holzer L. Treatment motivation in adolescents with psychosis or at high risk: Determinants and impact on improvements in symptoms and cognitive functioning, preliminary results. Psychotherapy Research. 2013; 23(4): 464-73.

137. Thomson A, Griffiths H, Fisher R, McCabe R, Abbott-Smith S, Schwannauer M. Treatment outcomes and associations in an adolescent-specific early intervention for psychosis service. Early Interv Psychiatry. 2019; 13(3): 707-14.

138. Higgins, Altman DG, Gøtzsche PC, Jüni P, Moher D, Oxman AD, et al. The Cochrane Collaboration's tool for assessing risk of bias in randomised trials. BMJ. 2011; 343: d5928.

139. Furukawa TA, Salanti G, Atkinson LZ, Leucht S, Ruhe HG, Turner EH, et al. Comparative efficacy and acceptability of first-generation and second-generation antidepressants in the acute treatment of major depression: protocol for a network meta-analysis. BMJ Open. 2016; 6(7): e010919.
